# Changes in Plasma Free Amino Acid Profile in Endurance Athletes over a 9-Month Training Cycle

**DOI:** 10.3390/metabo14070353

**Published:** 2024-06-23

**Authors:** Krzysztof Kusy, Monika Ciekot-Sołtysiak, Jan Matysiak, Agnieszka Klupczyńska-Gabryszak, Szymon Plewa, Ewa Anna Zarębska, Zenon J. Kokot, Paweł Dereziński, Jacek Zieliński

**Affiliations:** 1 Department of Athletics Strength and Conditioning, Poznan University of Physical Education, ul. Królowej Jadwigi 27/39, 61-871 Poznań, Poland; ciekot@awf.poznan.pl (M.C.-S.); zarebska@awf.poznan.pl (E.A.Z.); jzielinski@awf.poznan.pl (J.Z.); 2 Department of Inorganic and Analytical Chemistry, Poznan University of Medical Sciences, ul. Rokietnicka, 60-806 Poznań, Poland; jmatysiak@ump.edu.pl (J.M.); aklupczynska@ump.edu.pl (A.K.-G.); splewa@ump.edu.pl (S.P.); z.kokot@akademiakaliska.edu.pl (Z.J.K.); p.derezinski@gmail.com (P.D.); 3 Faculty of Health Sciences, Calisia University, ul. Nowy Świat 4, 62-800 Kalisz, Poland

**Keywords:** proteinogenic amino acids, non-proteinogenic amino acids, LC-ESI-MS/MS technique, training modality, progressive exercise, ammonia, lactate, maximum oxygen uptake, body composition

## Abstract

We aimed to evaluate long-term changes in proteinogenic and non-proteinogenic plasma free amino acids (PFAA). Eleven male endurance triathletes participated in a 9-month study. Blood was collected at rest, immediately after exhaustive exercise, and during 30-min recovery, in four consecutive training phases: transition, general, specific, and competition. Twenty proteinogenic and 22 non-proteinogenic PFAAs were assayed using the LC-ESI-MS/MS technique. The structured training modified the patterns of exercise-induced PFAA response, with the competition phase being the most distinct from the others. Branched-chain amino acids (*p* = 0.002; *η*^2^ = 0.216), phenylalanine (*p* = 0.015; *η*^2^ = 0.153), methionine (*p* = 0.002; *η*^2^ = 0.206), and lysine (*p* = 0.006; *η*^2^ = 0.196) declined more rapidly between rest and exhaustion in the competition phase. Glutamine (*p* = 0.008; *η*^2^ = 0.255), glutamate (*p* = 0.006; *η*^2^ = 0.265), tyrosine (*p* = 0.001; *η*^2^ = 0.195), cystine (*p* = 0.042; *η*^2^ = 0.183), and serine (*p* < 0.001; *η*^2^ = 0.346) levels were reduced in the competition phase. Arginine (*p* = 0.046; *η*^2^ = 0.138) and aspartate (*p* = 0.011; *η*^2^ = 0.171) levels were highest during exercise in the transition phase. During the competition phase, α-aminoadipic acid (*p* = 0.023; *η*^2^ = 0.145), β-aminoisobutyric acid (*p* = 0.007; *η*^2^ = 0.167), β-alanine (*p* < 0.001; *η*^2^ = 0.473), and sarcosine (*p* = 0.017; *η*^2^ = 0.150) levels increased, whereas phosphoethanolamine (*p* = 0.037; *η*^2^ = 0.189) and taurine (*p* = 0.008; *η*^2^ = 0.251) concentrations decreased. Overtraining indicators were not elevated. The altered PFAA profile suggests adaptations within energy metabolic pathways such as the tricarboxylic acid cycle, oxidative phosphorylation, ammonia neutralization, the purine nucleotide cycle, and buffering of intracellular H^+^ ions. The changes seem to reflect normal adaptations.

## 1. Introduction

Metabolic pathways involving amino acids are an integral and key area associated with the response to acute and chronic endurance exercise. Amino acids serve as essential substrates or intermediates in the tricarboxylic acid (TCA) cycle, glucose–alanine cycle, glutamine–glutamate cycle, urea cycle, purine nucleotide cycle, and aspartate–amine transport, among others [1,2,3]. Although they are not a direct source of energy during skeletal muscle activity, they are critical for maintaining the proper functioning of a variety of pathways. Circulating free amino acids, which represent a transient and small (~1% of total amino acids) but important reservoir of amino acids, can be referred to as a “metabolic currency” that is “traded” under conditions of stress such as exercise, starvation, or pathological states of the body [4,5]. 

Acute high-intensity exercise stimuli exceeding 70–75% of maximal oxygen uptake are required to significantly alter the concentration of free amino acids in the blood [6]. Another effective stimulus for pronounced changes in blood amino acid levels is prolonged (several hours) moderate-intensity exercise at or slightly above the ventilatory or lactate threshold [7]. Since both of these exercise modalities are commonly used by competitive and amateur endurance athletes, long-term alterations in blood amino acid profiles are to be expected. In fact, there are studies supporting this claim. However, they usually address relatively short training periods of 9 days to 10 weeks [8,9,10,11,12,13], and much less often, long periods of 5–10 months [14,15,16]. A significant portion of these papers only provided resting measurements, not addressing the exercise-induced response [10,11,13,14,15,17]. In addition, a limited set of free amino acids in the blood were usually analyzed [8,9,11,12,13,14,16]. Moreover, only one of the studies with multi-month cycles involved endurance-type training (swimmers) [16]. The other two long-term studies analyzed “mixed” training, i.e., including strength, speed, and other stimuli on par with endurance training in soccer players [14] and army recruits [15]. An important issue is the periodization of training, which is based on the deliberate control of the ratio between volume and intensity of loads in the main phases of training, sub-phases, macro- and micro-cycles [18]. Measuring the amino acid profile can help to better monitor adaptive changes and by extension the effectiveness of a periodized training program.

Importantly, only single non-proteinogenic amino acids have been included in a few studies dedicated to sports (or military) training [10,11,14]. Non-proteinogenic amino acids are not encoded by the genome or incorporated into proteins during translation. Those found in proteins are formed by post-translational modification during protein synthesis. They serve as intermediates in biosynthesis and perform various physiological functions during exercise metabolism, e.g., increasing energy expenditure or acting as potential myokines that are secreted from exercising skeletal muscle, and they affect other organs. They often play the role of intermediates in the metabolic pathways involving proteinogenic amino acids, e.g., ornithine and citrulline occur in the urea cycle [19]. There is a lack of data on the effects of long-term training on non-proteinogenic amino acid levels and their potential role as specific biomarkers of training status in athletes.

The within-subject biological variation in plasma amino acid concentrations appears to be relatively small [20], implying that under steady-state conditions, amino acid concentrations are stable. Since the studies cited above and many others have shown that the concentration of individual amino acids or groups of amino acids in the blood may change in response to specific exercise or training modality, the profile of plasma free amino acid (PFAA) can be used as a diagnostic tool to assess the status of metabolic adaptation. PFAA levels and patterns of change may provide a baseline for assessing training effects. PFAA profile offers information on the intensity of amino acid transfer between organs, allowing indirect inferences about the metabolic processes taking place and their nature. Our research may fill a gap in the research of athletes during long training cycles. Therefore, the aim of this study was to evaluate changes in the plasma profile of a wider range of proteinogenic and non-proteinogenic amino acids in endurance athletes during a long training cycle structured according to the principles of periodization in competitive sports [18]. We hypothesize that the PFAA profile will change during the major training phases as a result of metabolic adaptations, i.e., we expect that the PFAA levels or patterns of their exercise-induced response will be modified.

## 2. Materials and Methods

### 2.1. Participants

Eleven male endurance athletes (triathletes) competing at national and international levels, aged 24.6 ± 5 years, height 182 ± 5 cm, participated in this study. They were recruited from one professional sports team and had been involved in competitive endurance sports for at least 5 years. They were specialized in the standard Olympic distance (1.5 km swimming, 40 km cycling, 10 km running). They underwent regular medical examinations as recommended by the relevant sports federation. They were in good health and free of injury during the study period. In addition, they had never tested positive in an anti-doping test. Study procedures adhered to the Helsinki Declaration of 1964 and subsequent amendments. This study was approved by the Bioethics Committee of the Poznan University of Medical Sciences (decision no. 1252/18 of 6 December 2018). The purpose, risks, and benefits of the study were explained to the athletes. The athletes signed a written informed consent form prior to the start of the study.

### 2.2. Study Design

**Figure 1:**
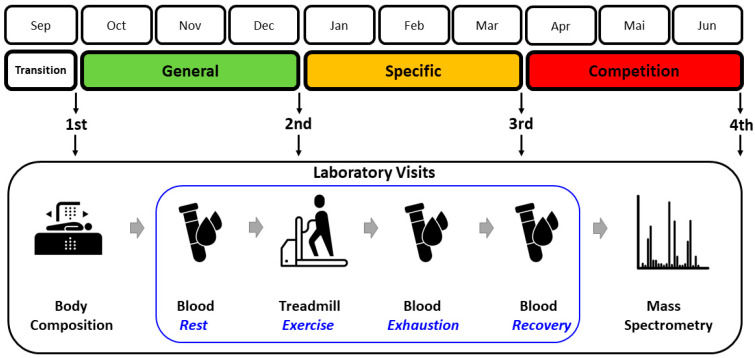
Study design. Adaptation of the four examinations to the consecutive training phases and the flow of the main measurement procedures. Terms of use: The icons used in this figure are licensed under CC BY 3.0 license. They are attributed to Luis Prado (body composition), Lars Meiertoberens (blood), Rafiico Creative Studio (treadmill), and Fredrik Edfors (mass spectrometry). The original versions can be found at https://thenounproject.com (accessed on 24 May 2024).

The testing procedures were adapted to the athletes’ 9-month training cycle (Figure 1). There were four data collection points at the end of major training phases: (i) after a 1-month transition (detraining) phase, (ii) after a 3-month general preparation phase, (iii) after a 3-month specific preparation phase, and (iv) after 3 months of pre-competition (tapering) and competition season. During the transition phase, training loads were minimized and athletes focused on physical recovery. The general preparation phase was characterized by a predominance of low- to moderate-intensity, high-volume aerobic exercise. During the specific preparation phase, the amount of low- to moderate-intensity training loads was maintained, but the proportion of high-intensity anaerobic loads was increased. In the pre-competition and competition periods, the volume of moderate-intensity aerobic loads substantially decreased, while the proportion of high-intensity anaerobic loads continued to increase. At the end of each phase, the athletes underwent the measurements described in the following subsections.

### 2.3. Testing Protocol 

The exercise tests were conducted at the Human Movement Laboratory ‘LaBthletics’ at the Poznan University of Physical Education, Poznan, Poland. The athletes refrained from high-intensity or prolonged exercise 24–48 h prior to the laboratory visit. Throughout the study, the participants maintained their usual dietary habits and intake of supplements. The latter included standard preparations used in sports, containing vitamins, micronutrients, carbohydrates, isotonic fluids, β-alanine (2/11 athletes), and branched-chain amino acids (6/11 athletes). The consumption was not programmed or controlled by our team quantitatively or qualitatively, so the athletes pursued their individual habitual routines. Preparation for laboratory visits was standardized to minimize the impact of ad hoc confounders. Prior to the day of testing, participants were instructed to avoid excessive fluid intake or dehydration, and to refrain from taking any supplements or aids that might directly affect their PFAA levels or physical performance. The athletes arrived at the lab in the morning after an overnight fast of approximately 12 h. During the laboratory visit, a 48 h dietary record was reviewed to identify any abnormal eating or supplementation patterns that might affect the measurements. Before starting the procedures, the participants urinated and had a bowel movement.

Height and weight were measured using a SECA 285 stadiometer (SECA GmbH, Hamburg, Germany). Body composition, including fat mass, lean body mass, and related indicators, was determined using the dual X-ray absorptiometry (DXA) method (Lunar Prodigy device and enCORE software, version 17.50.037; GE Healthcare, Chicago, IL, USA), as previously described [21]. In addition, skeletal muscle mass (SMM) was estimated using the prediction equation by Kim et al. [22].

A graded exercise test was performed on the h/p Cosmos Pulsar treadmill (Sports & Medical GmbH, Nussdorf-Traunstein, Germany). Breath-by-breath gas exchange measurements were continuously recorded with the MetaLyzer 3B ergospirometer, and the raw data obtained were processed with the MetaSoft Studio 5.1.0 software package (Cortex Biophysik GmbH, Leipzig, Germany). Heart rate was monitored with the Polar Bluetooth Smart H6 monitor (Polar Electro Oy, Kempele, Finland). For the purpose of this study, the most important variable was maximal oxygen uptake ($\dot{V}$O_2max_). Prior to the test, the athlete remained stationary for 3 min to ensure proper functioning of the measurement system. The speed was then increased to 4 km⋅h^−1^ and after another 3 min to 8 km⋅h^−1^. For the remainder of the test, the speed was increased by 2 km⋅h^−1^ every 3 min until exhaustion. The total exercise time was approximately 20 min. After completing the exercise, the athlete walked at a speed of 4 km⋅h^−1^ for another 3 min and then rested in a seated position for 30 min while waiting for the next blood sample. The laboratory temperature was maintained at a constant 20–21 °C.

Blood was collected four times: before the test under resting conditions, then immediately after the test at the time of exhaustion, and finally twice during the post-exercise recovery period (15 and 30 min after completion of the test). Blood samples (2.5 mL each) were obtained using a peripheral venous catheter placed in the antecubital vein. Plasma separation tubes (containing EDTA) were used to collect samples, which were centrifuged at 13,000 rpm for 3 min at 4 °C (Universal device, Hettich GmbH, Tuttlingen, Germany). Plasma was then pipetted into 0.5 mL vials and immediately frozen in liquid nitrogen. The vials were stored in the HEF^®^ U410 (New Brunswick Scientific Co., Inc., Edison, NJ, USA) low-temperature freezer at −80 °C until further PFAA analysis. Some other metabolites and blood morphotic elements were determined immediately. Blood lactate and ammonia concentrations were measured using the Biosen C-line (EKF-diagnostic GmbH, Barleben, Germany) and PocketChem BA (Arkray Inc., Kyoto, Japan) devices, respectively. Resting creatine kinase levels were measured with the Reflotron Plus device (Roche Diagnostics International AG, Basel, Switzerland). White and red blood cells, hemoglobin, and hematocrit were measured using the Sysmex XS-1000i (Sysmex Europe, Hamburg, Germany).

### 2.4. PFAA Analysis

Plasma samples for the PFAA assay were analyzed at the Department of Inorganic and Analytical Chemistry at the Poznan University of Medical Sciences, Poznan, Poland. The liquid chromatography electrospray ionization tandem mass spectrometry (LC-ESI-MS/MS) technique and the aTRAQTM reagent (Sciex, Framingham, MA, USA) were used. This method has high specificity, accuracy, and precision for the quantification of PFAA concentrations [23]. The suitability of the system was tested before each sequence to check the reproducibility of the retention time, the intensity of the internal standard peaks (instrument sensitivity), and the overall performance of the LC-ESI-MS/MS. Samples were randomly prepared and analyzed to avoid bias in the results. All PFAA concentrations were corrected for changes in plasma volume relative to resting values [24]. Detailed procedures and lower limits of quantitation for each amino acid are presented in Appendix A.

A total of 42 PFAAs and related metabolites were analyzed. Twenty proteinogenic PFAAs were included: L-histidine (His), L-isoleucine (Ile), L-leucine (Leu), L-lysine (Lys), L-methionine (Met), L-phenylalanine (Phe), L-threonine (Thr), L-tryptophan (Trp), L-valine (Val), L-arginine (Arg), L-cystine (Cyss), L-glutamine (Gln), glycine (Gly), L-proline (Pro), L-tyrosine (Tyr), L-alanine (Ala), L-asparagine (Asn), L-aspartic acid (Asp), L-glutamic acid (Glu), and L-serine (Ser). Sixteen non-proteinogenic amino acids were considered: 1-methyl-L-histidine (1MHis), 3-methyl-L-histidine (3MHis), L-α-amino-adipic acid (Aad), L-α-amino-n-butyric acid (Abu), D,L-β-aminoisobutyric acid (bAib), β-alanine (bAla), L-citrulline (Cit), hydroxy-L-proline (Hyp), L-ornithine (Orn), sarcosine (Sar), taurine (Tau), L-argininosuccinic acid (Asa), cystathionine (Cth), L-homocitrulline (Hcit), γ-amino-n-butyric acid (GABA), and δ-hydroxylysine (Hyl). Six related metabolic products completed the above set: ethanolamine (EtN), O-phospho-ethanolamine (PEtN), L-anserine (Ans), L-carnosine (Car), L-homocystine (Hcy), and O-phospho-L-serine (PSer). In addition, the Phe/Tyr ratio was calculated as an indicator of the catabolic state associated with injury and infection [17], and the Gln/Glu ratio was used to assess overall training tolerance and potential overreaching [11,25].

### 2.5. Statistics

We used Python 3.12.2 and several scientific libraries to perform the t-distributed stochastic neighbor embedding (t-SNE) analysis and visualize the clustering of multidimensional data (149 variables) that described each athlete in each training phase. Key packages included pandas for data manipulation, scikit-learn for implementing the t-SNE algorithm, matplotlib for creating the visualizations, and numpy for numerical computations. The t-SNE parameters were selected to ensure optimal visualization: a moderate perplexity of 20 was used to balance local and global data structure due to the limited sample size of study participants, and a learning rate of 200 and 5000 iterations were used to ensure proper convergence of the algorithm. The concentrations of individual PFAAs and the values of other variables were expressed as means and standard deviations. Parametric tests were used for detailed analyses, as the Shapiro-Wilk test showed a normal distribution of the data for the main variables analyzed. A two-way repeated measures analysis of variance (ANOVA) was used to assess the effects of training phase, exercise test stage, and their interaction on the concentration of each PFAA. Assuming 4 measurements in consecutive training phases, 4 sampling points during the exercise test, a *p*-value of 0.05, a statistical power of 0.8, and a partial *η*^2^ of 0.14, a minimum number of subjects of 10 was determined using G*Power 3.1.9.6 software [26]. Levene’s test was used to test for the equality of variance. Mauchly’s test was used to assess sphericity, followed by the Greenhouse-Geisser correction if sphericity was violated. The Bonferroni correction was applied as a post-hoc test when main effects or interactions showed significance. Partial *η*^2^ was used as a measure of ANOVA effect size (small 0.01, medium 0.06, large 0.14). The significance level was set at *p* ≤ 0.05 for all analyses. The original raw, unadjusted data used for the calculations are available as Supplementary File S1. 

## 3. Results

### 3.1. Descriptive Characteristics

Changes in basic somatic, hematological and cardiorespiratory indices are shown in Table 1. Both fat mass and fat percentage decreased significantly between the transition and general phases and then remained at similar levels until the competition phase. The same pattern of change was observed for percent muscle mass, while muscle mass in kilograms remained virtually constant throughout the study period. Resting hemoglobin concentration remained at similar levels during the first three phases and decreased significantly during the competition phase. Maximum oxygen uptake per kg body weight gradually increased from the first to the last test, reaching a statistically significant difference between the transition and competition phases. Absolute (L per minute) and SMM-adjusted $\dot{V}$O_2max_ values did not change significantly. The maximum heart rate showed a downward trend between the transition and competition phases. Body weight, body mass index, white and red blood cell count, hematocrit, and creatine kinase levels did not change significantly during the training cycle studied despite some slight trends. 

### 3.2. Clustering Multidemensional Data

**Figure 2:**
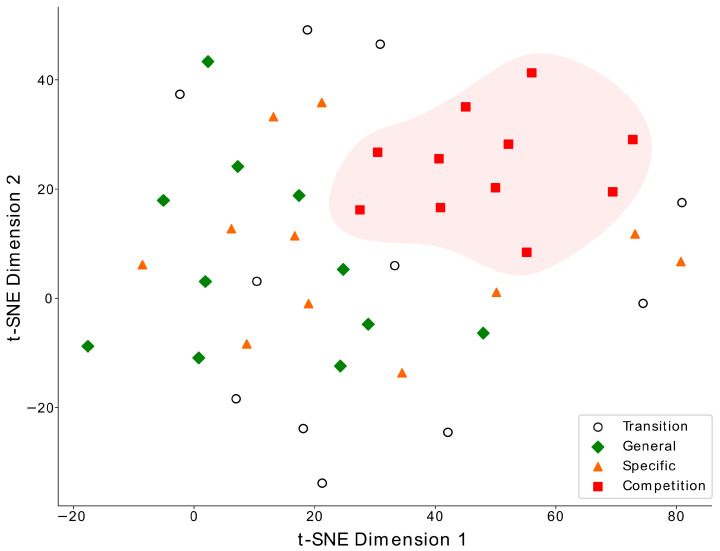
The t-SNE visualization of the multidimensional data of the athletes from four training phases: transition (open circles), general (green diamonds), specific (orange triangles), and competition (red squares). Each point represents one athlete in one training phase with a set of 149 characteristics (concentrations of plasma amino acids, lactate and ammonia, body composition, maximal oxygen uptake, creatine kinase, and blood count). The data from the competition phase form a distinct cluster.

As a result of the t-SNE analysis, the most notable dimensional clustering was observed in the competition phase, where the data points of all 11 study participants formed a distinct cluster, indicated by red squares in Figure 2, highlighting a specific metabolic state achieved during this training phase. Data from other training phases did not yield consistent clusters, although there were some weak tendencies to group fewer athletes.

### 3.3. Plasma Free Amino Acid Concentrations

**Figure 3:**
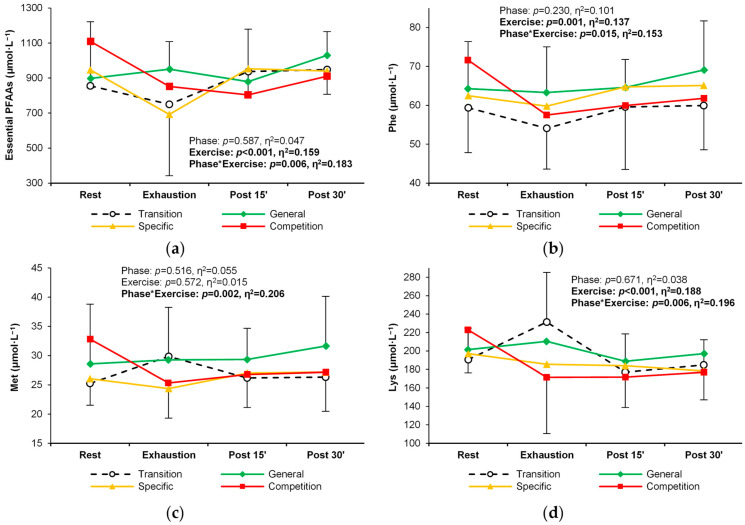
Change in the plasma concentration and exercise-induced response of three essential amino acids in the four training phases: (**a**) total essential amino acids, (**b**) phenylalanine, (**c**) methionine, and (**d**) lysine. Significant effects of the analysis of variance are shown in bold.

**Figure 4:**
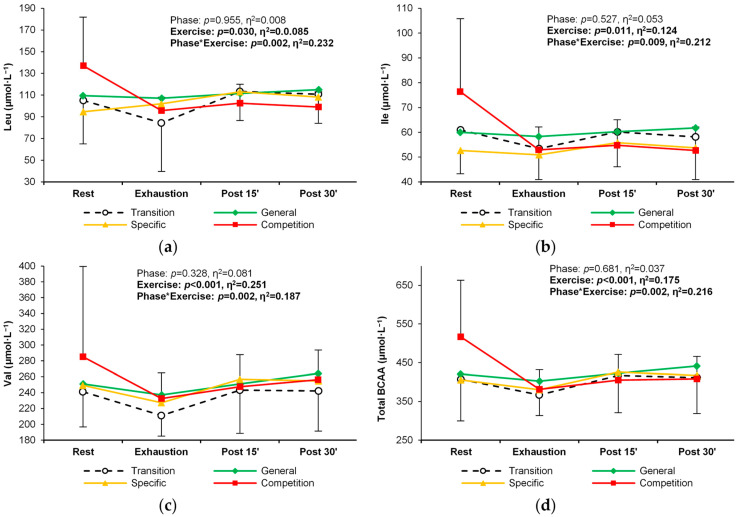
Change in the plasma concentration and exercise-induced response of branched-chain amino acids (BCAA) in the four training phases: (**a**) leucine, (**b**) isoleucine, (**c**) valine, and (**d**) combined BCAA. Significant effects of the analysis of variance are shown in bold.

Except for methionine, the concentration of essential PFAAs changed significantly in response to exercise (Table 2). In general, there was a decrease between rest and exhaustion, followed by an increase during recovery (Table 2, Figure 3a). For six individual essential PFAAs, a changing pattern of response to exercise was observed over successive training periods. Methionine, phenylalanine, and lysine decreased more rapidly between rest and exhaustion in the competition phase, with similar levels during recovery in all training phases. In addition, the lysine concentration was significantly higher at exhaustion in the transition phase (Table 2, Figure 3b–d). An analogous alteration in the exercise-induced response was observed for branched-chain amino acid (BCAA) concentrations (leucine, isoleucine, and valine) (Table 2, Figure 4a–d). 

**Figure 5:**
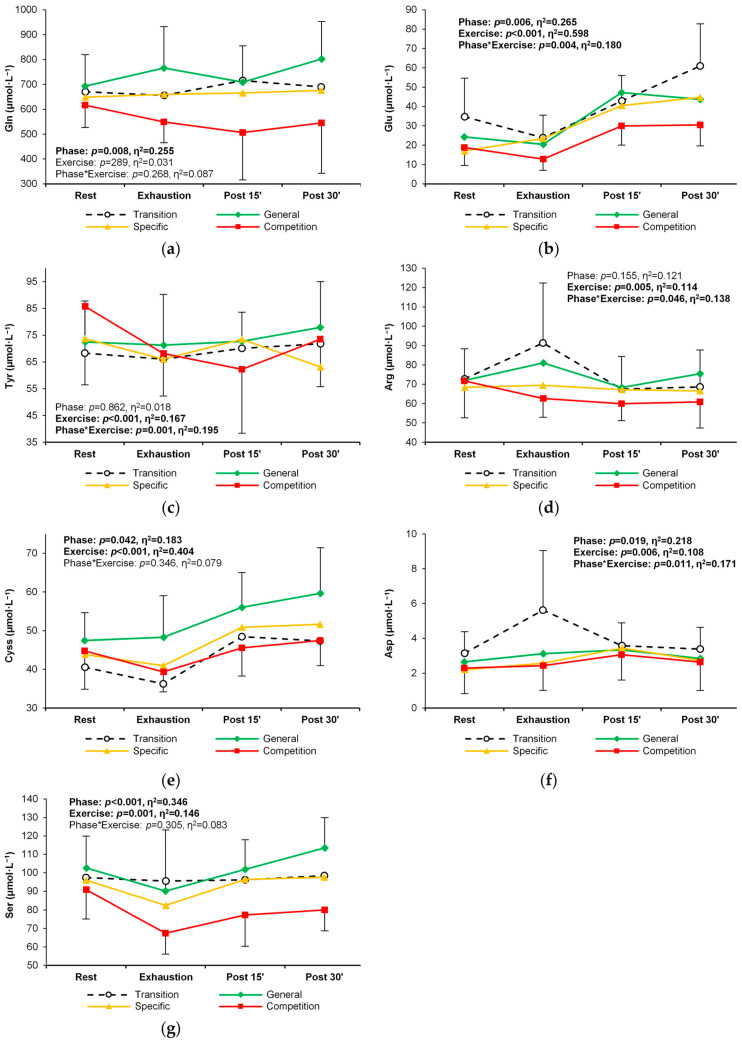
Change in the plasma concentration and exercise-induced response of seven non-essential amino acids in the four training phases: (**a**) glutamine, (**b**) glutamic acid, (**c**) tyrosine, (**d**) arginine, (**e**) cystine, (**f**) aspartic acid, and (**g**) serine. Significant effects of the analysis of variance are shown in bold.

A significant response to exercise was observed for almost all non-essential PFAAs, with the exception of glutamine. The general trend for this PFAA group was an increase in concentration from rest, through exhaustion, until the 30-min recovery (Table 3). During the long training cycle analyzed, alterations in the levels or patterns of exercise-induced change were seen for seven non-essential amino acids (Table 3, Figure 5a–g). A reduction in the plasma concentrations of glutamine, glutamate, tyrosine, cystine, and serine was observed in the competition phase at rest, during exercise, and post-exercise recovery. Also, higher arginine (Figure 5d) and aspartic acid (Figure 5f) levels at exhaustion were revealed in the transition phase compared to other phases.

**Figure 6:**
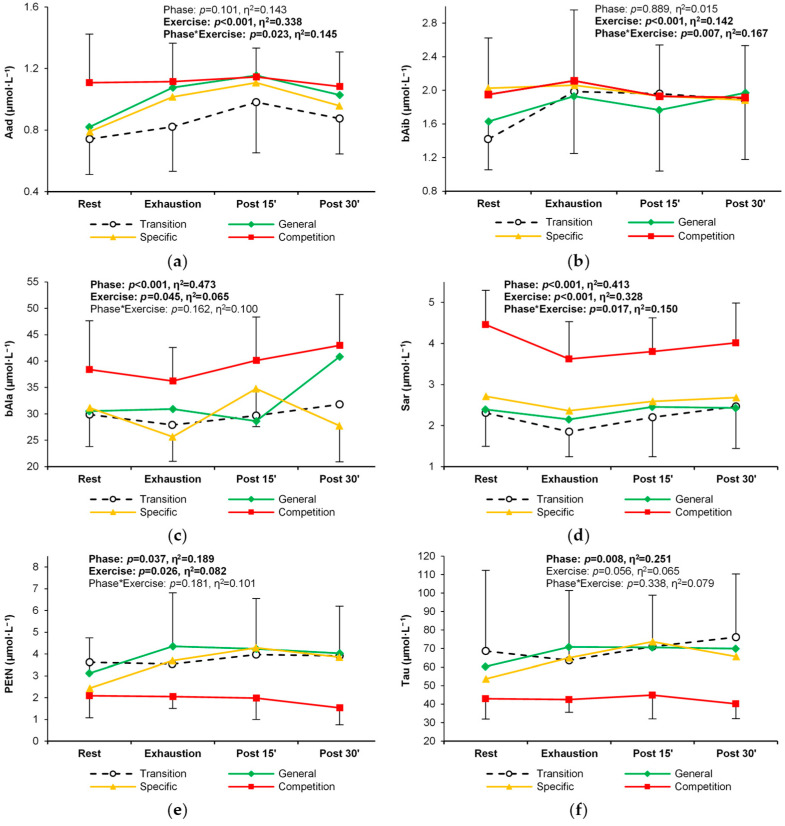
Change in the plasma concentration and exercise-induced response of non-proteinogenic amino acids in the four training phases: (**a**) amino-adipic acid, (**b**) β-aminoisobutyric acid, (**c**) β-alanine, (**d**) sarcosine, (**e**) phospho-ethanolamine, and (**f**) taurine. Significant effects of the analysis of variance are shown in bold.

**Figure A1:**
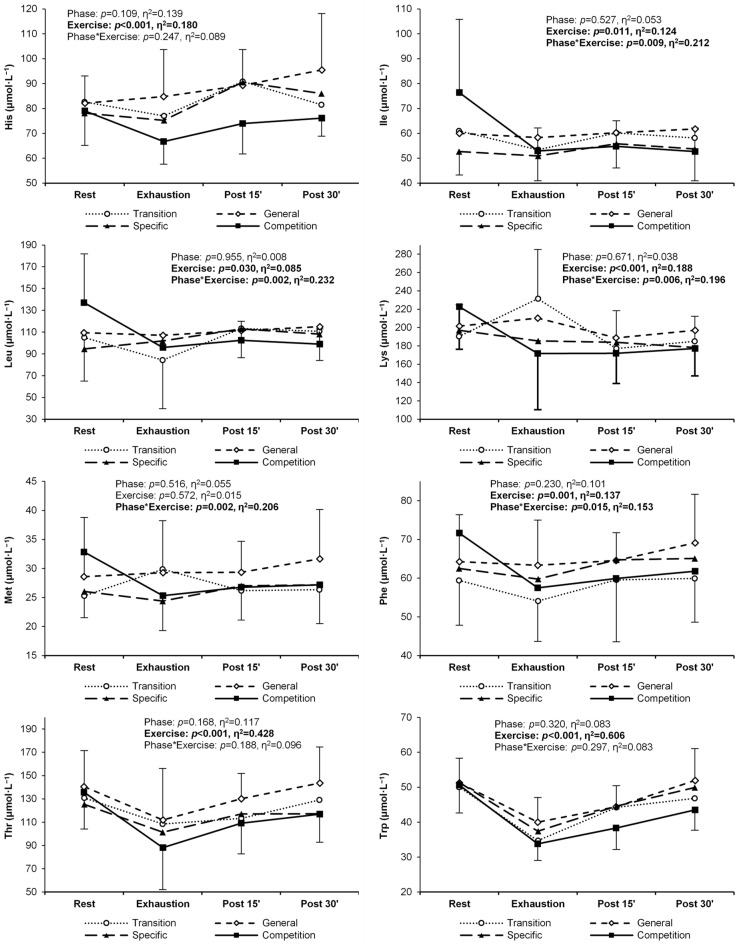

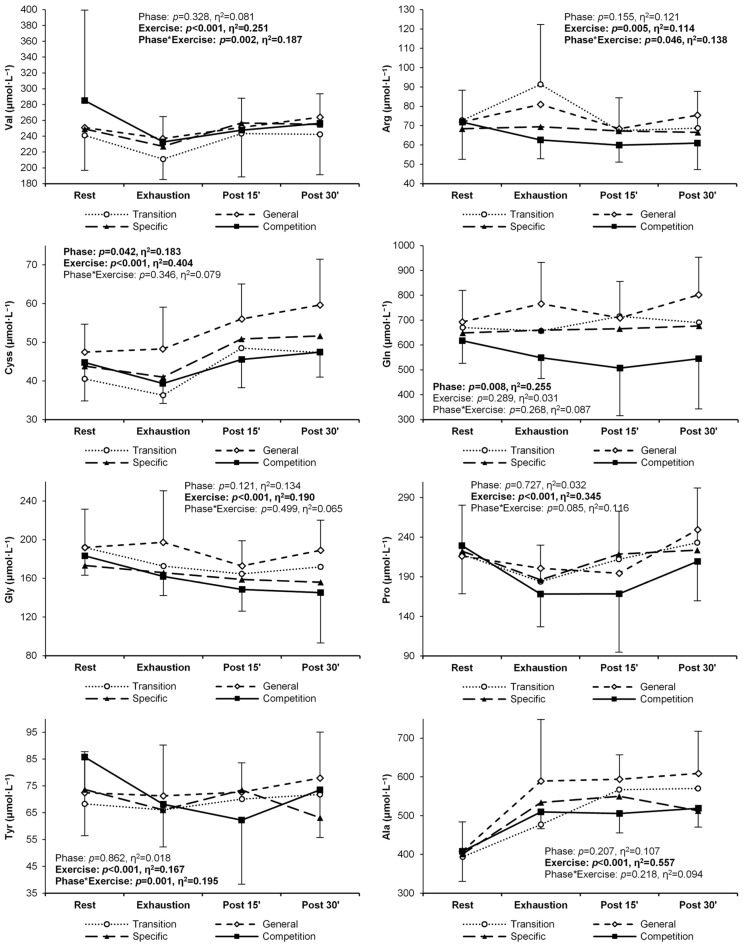

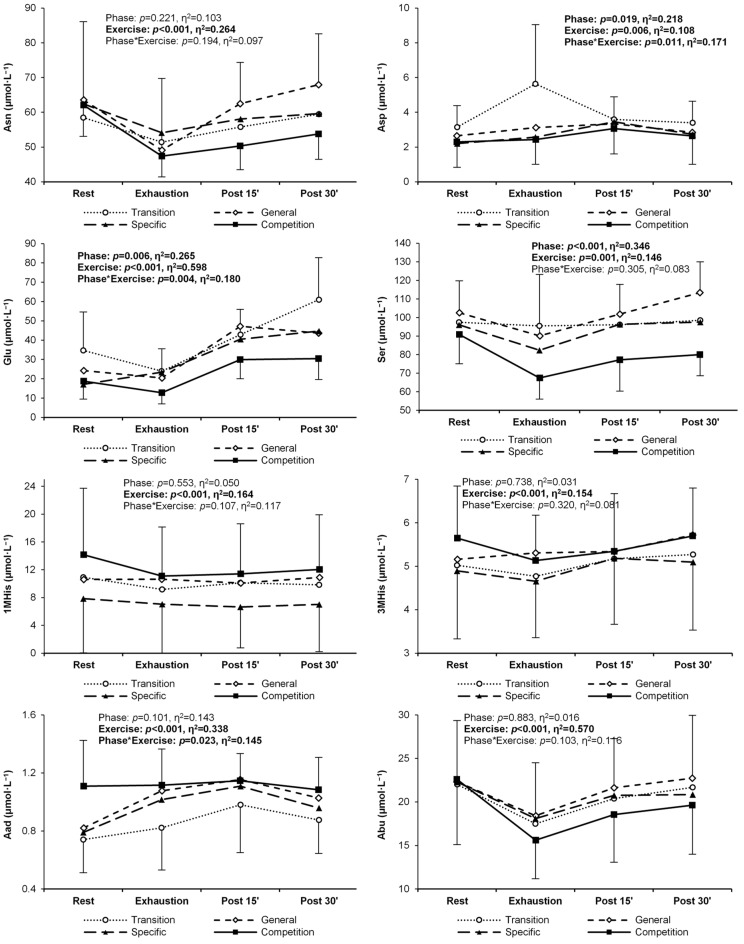

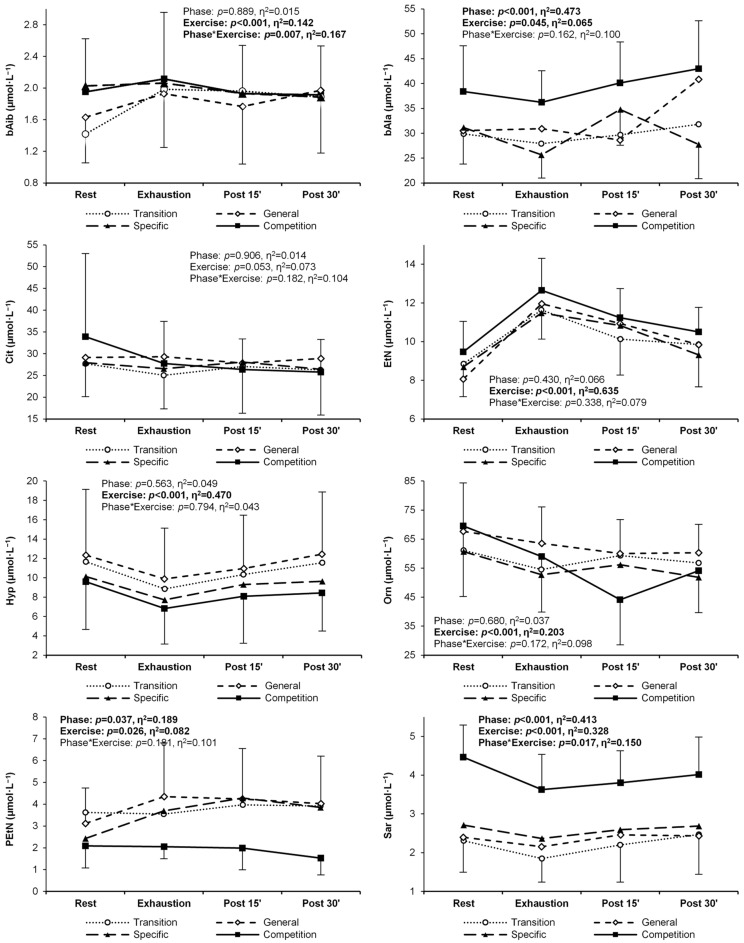

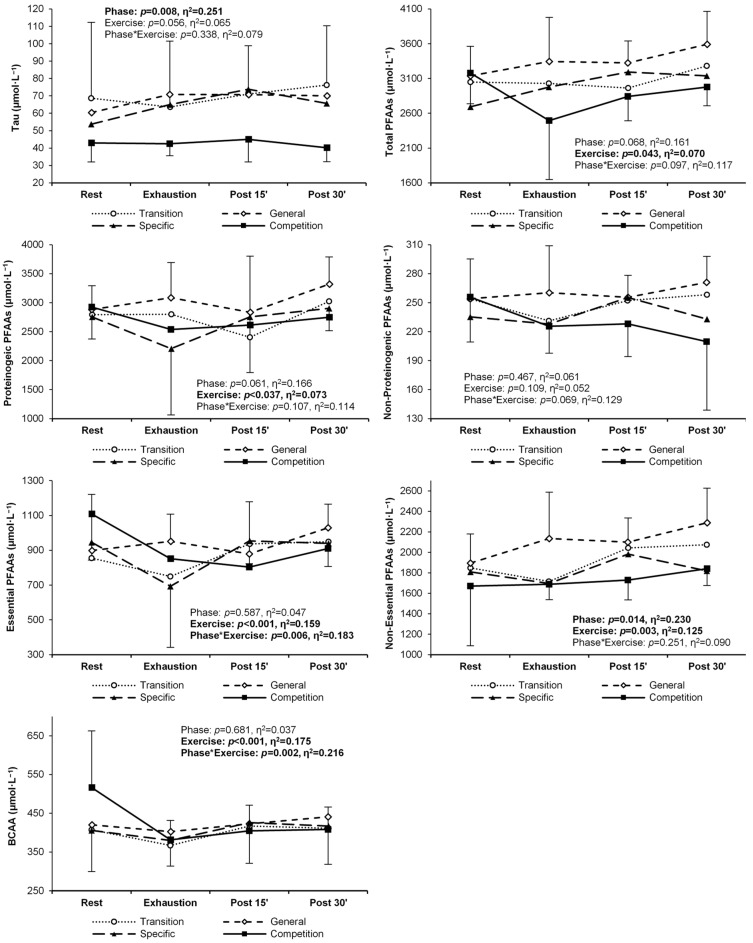
Plasma concentrations of plasma free amino acids in endurance athletes at rest, under exercise-induced exhaustion, and during post-exercise recovery in the four main training phases of a 9-month cycle. Significant ANOVA effects of the training phase, exercise stage, and their interaction are shown in bold. For amino acid abbreviations, see the main text (Materials and Methods, Section 2.4. PFAA Analysis).

Nine of the twenty-two non-proteinogenic PFAAs and related metabolites analyzed did not exceed the lower limit of quantitation. These were argininosuccinic acid, cystathionine, anserine, carnosine, homocitrulline, homocystine, phosphoserine, γ-aminobutyric acid, and δ-hydroxylysine. Eleven of 13 non-proteinogenic metabolites responded significantly to exercise. Concentrations of 1-methylhistidine, 3-methylhistidine, aminobutyric acid, β-alanine, hydroxyproline, ornithine, and sarcosine decreased with exercise, whereas concentrations of aminoadipic acid, β-aminoisobutyric acid, ethanolamine, and phosphoethanolamine increased (Table 4). Significant changes between training phases were observed for six non-proteinogenic amino acids. Amino-adipic acid, β-aminoisobutyric acid, β-alanine, and sarcosine showed the highest levels and different patterns of exercise-induced response during the competition phase (Table 4, Figure 6a–d). In contrast, phospho-ethanolamine and taurine concentrations were lowest in the competition phase (Table 4, Figure 6e,f). Figures illustrating changes in all proteinogenic and non-proteinogenic PFAAs are available in Appendix B (Figure A1).

### 3.4. Training Status Biomarkers

**Figure 7:**
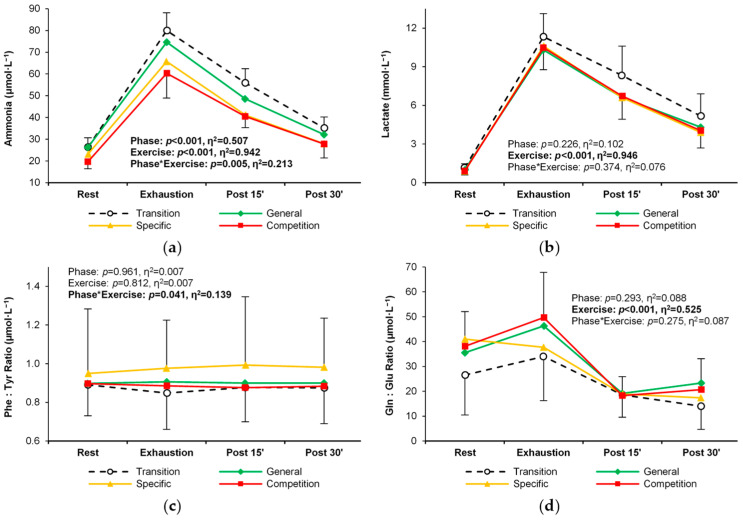
Change in the levels and exercise-induced response of training status biomarkers in the four training phases: (**a**) lactate, (**b**) ammonia, (**c**) phenylalanine: tyrosine ratio, and (**d**) glutamine: glutamate ratio. Significant effects of the analysis of variance are shown in bold.

Blood ammonia levels changed significantly during the subsequent training phases, with a significant and consistent decrease between the transitional and competition phases (Table 5, Figure 7a). The magnitude and pattern of exercise-induced changes in lactate, the phenylalanine/tyrosine ratio, and the glutamine/glutamate ratio were not significantly affected by the subsequent training phases (Table 5, Figure 7b–d). 

## 4. Discussion

**Figure 8:**
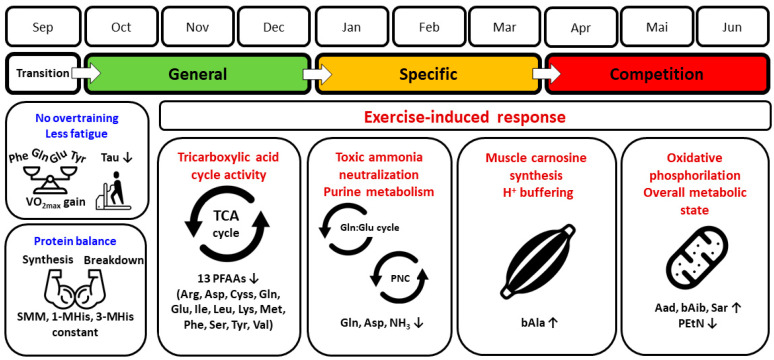
Normal adaptation response of plasma free amino acid concentration and related variables in competitive endurance athletes over a 9-month structured training period. Main significant changes between transition (lack of training) and competition (high-intensity, low-volume training) phases and potentially involved metabolic mechanisms are indicated. See the following sections for explanations. Abbreviations: 1-MHis, 1-methylhistidine; 3-MHis, 3-methylhistidine; Aad, amino adipic acid; Arg, arginine; Asp, aspartic acid; bAib, ß-aminoisobutyric acid; bAla, β-alanine; Cyss, cystine; Gln, glutamine; Glu, glutamic acid; H^+^, hydrogen ion; Ile, isoleucine; Leu, leucine; Lys, lysine; Met, methionine; NH_3_, ammonia; PEtN, phosphoethanolamine; PFAAs, plasma free amino acids; Phe, phenylalanine; PNC, purine nucleotide cycle; Sar, sarcosine; Ser, serine; SMM, skeletal muscle mass; Tau, taurine; TCA cycle, tricarboxylic acid cycle; Tyr, tyrosine; Val, valine; $\dot{V}$O_2max_, maximal oxygen uptake; ↓, decrease; ↑, increase. Terms of use: The icons used in this figure are licensed under CC BY 3.0 license. They are attributed to IYIKON (scale), Rafiico Creative Studio (treadmill), Uswah Studio (biceps), Hrbon (cycles), Brickclay (muscle), and Andi Nur Abdillah (mitochondrion). The original versions can be found at https://thenounproject.com (accessed on 24 May 2024).

The main finding of our study is that over a 9-month period, significant alterations in the PFAA profile occurred in endurance athletes between typical major training phases: from detraining, through general and specific preparation, to competition. As the global t-SNE analysis showed, the competition phase was the most distinct from the other phases, indicating the achievement of specific and targeted metabolic adaptations in this final stage of the training cycle. At the individual amino acid level, long-term training substantially affected both proteinogenic and non-proteinogenic amino acids and related metabolites by modifying their plasma concentrations or patterns of response to graded maximal exercise. The main significant directions of change and potentially involved mechanisms are summarized in Figure 8 and discussed in further paragraphs.

We showed that the consecutive 3-month phases induced significant training-specific changes in the PFAA profile. We were not able to determine the timing of the first significant change in the amino acid profile in each phase because it was not possible to test the athletes frequently, e.g., weekly or bi-weekly, for several months. Previous studies using short training cycles showed that 9-day and 2-week training programs do not cause significant changes in plasma tryptophan and BCAA levels [12,13], whereas 4–10 weeks of training modify BCAA, glutamine, arginine, tyrosine, tryptophan, serine, aspartate, glycine, phenylalanine, arginine, and taurine concentrations [8,9,10,11]. It appears that the first significant adaptation symptoms in the form of PFAA profile changes should be expected only after several weeks. Therefore, PFAA profile analysis may be a tool for the long-term, rather than ad hoc, diagnosis of an athlete. We assume also that the initial training status; the type, intensity, and volume of training loads; the particular amino acid; and other training-related characteristics determine the direction and rate of change in PFAA profiles. 

### 4.1. Normal or Abnormal Response?

An important question seems to be whether the observed changes are the result of normal training adaptation or rather a symptom of overtraining. Some studies suggest that substantial changes in amino acid blood concentration are due to overtraining [11,12,16,17,25]. On the other hand, there are also reports of no negative effect on amino acid levels in athletes exposed to training overload [13,27]. The concentration of PFAAs in our athletes did not differ significantly from the ranges shown in normally adapted high-performance athletes [17]. Thus, it appears that our athletes were not amino acid deficient throughout the study period. In addition, the phenylalanine/tyrosine and glutamine/glutamate ratios did not change significantly over the course of the 9 months analyzed and remained outside the unfavorable range [17,25], suggesting that potential processes associated with overtraining did not develop. Similarly, plasma levels of 1- and 3-methylhistidine, the breakdown products of contractile actin and myosin proteins [1], did not change significantly between the training phases, indicating a relatively constant protein degradation without an excessive increase in the rate of muscle protein degradation processes.

Equally important, other indicators of training status were at levels typical of normal adaptation in competitive endurance athletes. As far as resting characteristics are concerned, these were decreasing fat mass and constant muscle mass [28], some decrease in hemoglobin concentration in the competition phase [29], and relatively low creatine kinase levels [30]. In terms of key functional characteristics, $\dot{V}$O_2max_ tended to increase and maximum heart rate tended to decrease, even though a stabilization of these basic parameters is also normal in experienced competitive athletes [31,32]. It is also a common observation that there is only a slight change in the maximum exercise-induced lactate concentration and a significant decrease in the maximum blood ammonia concentration over longer periods in highly trained athletes [32]. Typically, the above changes are accompanied by other favorable adaptations, including biomarkers of energy metabolism, such as increments in erythrocyte adenylate energy charge and the adenosine triphosphate/adenosine diphosphate ratio, a decrease in hypoxanthine levels, and an increase in hypoxanthine-guanine phosphoribosyl transferase activity [31,33,34]. $\dot{V}$O_2_ kinetics during exercise may also be improved, despite the lack of an increase in $\dot{V}$O_2max_ [35]. Thus, it can be assumed that the changes we observed in PFAA concentration were the result of normal training adaptation.

### 4.2. Comparison with Other Long-Term Studies

There is a paucity of literature on PFAA changes over long training cycles in athletes. The study by Krause et al. [16] on swimming training in 15- to 18-year-old athletes seems to have the most comparable design, although only BCAA, glutamine, and glutamate concentrations were analyzed. Their study covered a training period of the same duration (9 months) with key phases: basic, high load, tapering, and recovery. No significant changes in psychological characteristics (activity, mood, physical condition) and immunological parameters were observed. Contrary to our results, they showed a slight decrease in plasma BCAA concentration in the tapering period compared to basic (general) and high-load (specific) phases. On the other hand, similar to our study, during tapering, there was a similar noticeable tendency for BCAAs to decline more rapidly between rest and peak exercise, indicative of increased BCAA extraction from blood as an adaptation. Also, glutamine, glutamate, and ammonia levels decreased, during tapering, as in our study. This supports the thesis that the (pre-)competition phase is metabolically different from other phases of a standard long training cycle. This phase is typically characterized by a significant reduction in total training load, especially due to volume reduction. Apparently, this change in load structure and exercise stimuli after a long period of greatly increased load results in the modification (optimization) of metabolic processes that are important for exercise capacity and athletic performance.

Gwin et al. [15] performed a global metabolomic study in army infantry recruits (aged 22 ± 3 years) before and after 5-month military training divided into basic combat (general conditioning) and individual (military-related activities) training; thus, the physical load structure, goals, and tasks differed from those typical of endurance sports. Resting serum concentration of BCAAs, histidine, fatty acids, and urea cycle metabolites increased significantly. Also, the levels of tryptophan, tyrosine, methionine, cysteine, and taurine metabolites increased more than two-fold. These increases were interpreted in terms of higher protein turnover and the resulting anabolic response of body composition, i.e., loss of fat mass and gain of fat-free mass, which were expected beneficial adaptations. Van den Baar et al. [14] followed changes in resting plasma concentration of 14 amino acids over a 10-month season in young soccer players (14–17 years old). Unfortunately, they did not identify and compare any specific training phases (measurements at fixed 1-month intervals), but instead calculated general linear time trends of PFAA levels. They found an overall decrease in taurine levels and an increase in lysine and ornithine levels over the course of the season. 

The three studies cited above, as well as ours, show that long-term training cycles in healthy highly trained individuals may cause significant changes in the profile of free amino acids in the blood. Given the differences in the results obtained, it is reasonable to assume that the magnitude and directions of changes in the PFAA profile are related to specific training goals and the type of adequately tailored exercise loads, not neglecting other important factors (e.g., age and biological maturity). Nevertheless, the “remodeling” of the amino acid profile appears to be a normal physiological adaptive response, as the athletes studied did not show overtraining symptoms. Therefore, changes in the levels of blood free amino acids can be considered interesting biomarkers to monitor the training status of the athlete.

### 4.3. Metabolic Context

#### 4.3.1. Proteinogenic PFAAs

The TCA cycle is a key process for the energy metabolism of exercising muscles. In principle, all proteinogenic amino acids are involved in the TCA and enter it as carbon skeletons in so-called anaplerotic reactions, providing key intermediates (α-ketoglutarate, succinyl-CoA, fumarate, and oxaloacetate), or are converted to pyruvate or acetyl-CoA [1,2,36]. Thus, it seems that higher plasma concentrations of PFAAs (implying their greater availability to skeletal muscles and other tissues) should be associated with greater efficiency of energy metabolic processes, including promoting higher concentrations of intermediates, a smoother TCA cycle, and more efficient acetyl-CoA oxidation. This claim is supported by studies that found substantially higher amino acid concentrations in both muscle and plasma in athletes than in untrained individuals [37,38]. However, such an overly simplistic interpretation may be misleading in the context of changes in resting and post-exercise PFAA levels over a long-term training cycle in highly trained individuals. First, in our endurance athletes, the changes in resting concentrations of any PFAA between the detraining and competition phases did not reach the level of significance, despite a weaker trend toward an increase in some proteinogenic amino acids (BCAAs, lysine, methionine, phenylalanine, tyrosine). Thus, this does not conclusively support the thesis that resting PFAA levels increase with the training status. Second, it is the change in PFAA response to exercise that seems more relevant in assessing adaptive changes and training status. In our study, we observed a significant change in the pattern and magnitude of response to exercise between the detraining and competition phases in most proteinogenic PFAAs. There was a trend for 13 of 20 PFAA to decline more rapidly between rest and exhaustion, resulting in persistently lower levels at exhaustion and during recovery. Also, in the case of the remaining seven PFAAs, for which statistically significant changes were not detected, an analogous but less pronounced tendency was observed (histidine, threonine, tryptophan, glycine, proline, alanine, asparagine). Our results therefore suggest that normal long-term training adaptation is not just a simple quantitative shift in the concentration of PFAAs (increasing their availability to the TCA cycle), but rather a more efficient extraction of circulating PFAAs during exercise.

In the competition phase, we observed the lowest plasma glutamine levels, in particular during exercise and recovery, and lower alanine level (statistically nonsignificant). These two amino acids play a central role in the process of ammonia neutralization as non-toxic nitrogen carriers, which are synthesized de novo in skeletal muscle using BCAAs, asparagine, aspartate, and glutamate, the latter constantly taken up from the circulation [36]. Again, an increase could be expected in training periods characterized by readiness for the highest sports achievements, which also means the highest ability to neutralize exercise-induced ammonia. The decline in plasma glutamine (as well as alanine and other PFAAs to some extent) can be interpreted as part of an economization process, associated with the increasing contribution of high-intensity loads and reduction of training volume during the pre-competition (tapering), and competition phases. Previously, it was demonstrated that high-intensity interval training results in decreased resting adenosine triphosphate (ATP) and total adenine nucleotide levels in skeletal muscle, accompanied by less ATP depletion during exercise [39,40]. In line with these observations, as our and other studies show, the levels of metabolites produced in the ATP breakdown pathway, including ammonia, gradually decreae between the detraining and competition phases in highly trained athletes, both practicing sprint and endurance disciplines [32,34]. A potential mechanism for this beneficial adaptation involves an increase in the activity of the hypoxanthine guanine phosphoribosyl transferase (HGPRT), which enables the recovery of inosine monophosphate (IMP) from hypoxanthine via a less energetically expensive “rescue” pathway. In further transformations, IMP is converted to adenosine monophosphate (AMP) in the purine nucleotide cycle (PNC), and then to adenosine diphosphate (ADP) and ATP [34]. In the PNC, the amino acid aspartate is active, the concentration of which was significantly increased at exhaustion in the detraining phase and low in the remaining phases in our athletes, which indicates a regulatory effect of training stimuli.

#### 4.3.2. Non-Proteinogenic PFAAs

The concentrations of 6 of 13 detected non-proteinogenic PFAAs showed significant alterations between the training phases. Beta-alanine, α-aminoadipic acid, ß-aminoisobutyric acid, and sarcosine tended to increase, whereas taurine and phosphoethanolamine tended to decrease during the competition phase.

Beta-alanine, together with histidine, is the precursor of carnosine, a dipeptide synthesized in human muscle tissue, and its derivatives (e.g., anserine). One of its functions is buffering intracellular H+ ions in skeletal muscle cells, produced during anaerobic glycolysis that is related to high-intensity exercise [41]. Beta-alanine is the rate-limiting precursor of carnosine, synthesized in the liver and available via the circulatory system [42,43]. The concentrations observed in the athletes we studied were many times higher than those typical of the healthy general population, with levels of a few micromoles per liter in the latter group [44]. Increased physical activity is known to be associated with increased ß-alanine concentrations [45]. In our athletes, the unusually high plasma β-alanine concentration, especially during the competition phase, may be indicative of both the greater endogenous hepatic synthesis induced by extremely high training loads and the supplementation with β-alanine; however, we do not know how much each of these factors contributed. Histidine is not synthesized in the human body; thus, its plasma concentration is dependent on its availability in food, apart from other factors. Interestingly, in all four training phases, the resting histidine levels were very similar (average values of ~78–83 μmol·L^−1^). This means that no factor significantly affected the resting concentration of histidine over the study period, including dieting and supplements. However, at exhaustion, a certain divergence in trends was observed. In the competition phase, the concentration decreased to ~67 μmol·L^−1^ (by ~13 μmol·L^−1^ or ~11%), whereas in the preceding training phases, the maximum concentration remained relatively stable compared to rest (~75–85 μmol·L^−1^). We can speculate that since the baseline values were virtually the same across the training phases (meaning the same availability in the circulatory system), the slight but noticeable decrease between rest and exhaustion in the competition phase was most likely due to intensified and more effective histidine extraction from blood, which supports the higher demand for and utilization of muscle carnosine synthesis. This could be considered one of the beneficial metabolic adaptations. As was previously demonstrated, sprint training increases skeletal muscle carnosine content, resulting in improved contractile activity due to better pH buffering and Ca^2+^ sensitivity [41]. Admittedly, our athletes were not sprinters, but the proportion of high-intensity exercise was increased during the competition phase (accompanied by the reduction of exercise volume), most likely inducing adequate adaptations.

Beta-aminoisobutyric acid is a myokine produced by skeletal muscle during exercise in the process of mitochondrial valine oxidation. It affects other tissues in an endocrine manner, e.g., osteocytes, adipose tissue, or beta-oxidation of hepatic fatty acids. Its activity is inversely correlated with cardiometabolic risk factors, insulin resistance, and atherosclerosis [2,46]. Plasma α-aminoadipic acid is an intermediary biomarker of lysine and tryptophan metabolism, strongly associated with the risk of developing diabetes and elevated for many years prior to the onset of this disease [47]. Therefore, increasing levels of these two PFAAs over the long training cycle can be considered an expression of the athletes’ improved metabolic state. Direct links to physical training and athletic performance cannot be ruled out, but further research is needed.

The metabolism of sarcosine and other amino acids (glycine, serine, and ethanolamine) is indirectly involved in the creatine cycle and the mitochondrial respiratory chain [48,49]. Available studies suggest that sarcosine may serve as a marker of overtraining, as short (several weeks) and physically intense training periods can lead to elevated serum sarcosine levels, along with metabolites such as peroxides, cortisol, and hypoxanthine [50]. However, resting plasma sarcosine concentrations in our athletes generally did not exceed population norms (typically 0–5 μmol·L^−1^) during successive training phases, despite elevated levels during the competition phase. Moreover, beneficial adaptations occurred in our participants as described in the previous paragraphs. Reference sarcosine levels for athletes are not available, and typical metabolite concentrations in athletes often differ significantly from those found in the general population. 

Phosphoethanolamine (which chemically is not an amino acid, but a phosphoric acid ester) is an ethanolamine derivative and a precursor of the phospholipid phosphatidylethanolamine, which is typically found in the inner leaflet of the membranes of all cells, including muscle cells, and is found in abundance in mitochondria [51]. The role of phosphatidylethanolamine includes, inter alia, stimulating oxidative phosphorylation activity. Research on cell lines revealed that reducing mitochondrial phosphatidylethanolamine impaired respiratory capacity, adenosine triphosphate production, and activities of electron transport chain complexes I and IV [52]. The role of phosphoethanolamine and related molecules in exercise and training is not clearly defined but deserves attention.

The largest amounts of taurine are present in skeletal muscle, but also in other organs (liver, kidney, brain) and plasma. It is the end product of the metabolism of the sulphur-containing amino acids cysteine and methionine. It fulfills many useful metabolic functions, e.g., glucose and lipid regulation, energy metabolism, anti-inflammatory modulation, antioxidant actions, and many others. Its release into plasma is directly proportional to exercise intensity and is likely due to taurine role in the regulatory mechanism of calcium ions (Ca^2+^) handling during muscle contraction [53]. However, in our endurance athletes, plasma taurine levels did not significantly change during exercise and were lowest in the competition phase. This may be surprising because much higher taurine concentrations are observed in oxidative (type I) than glycolytic (type II) muscle fibers [54]. Type I makes up a significantly larger percentage of muscle fibers (vastus lateralis) in endurance than non-endurance athletes, with a combination of age, gender, training volume, and sports performance level accounting for ~11% of the variance, apart from genetic factors [55]. Consequently, increased release from muscle and higher plasma taurine levels can be expected after exhaustive exercise during the competitive phase. On the other hand, it is suggested that the increase in plasma taurine concentration after strenuous exercise may indicate, among others, muscle damage or fatigue [56], and elevated plasma taurine levels have been found in catabolic diseases [57]. From this point of view, reduced plasma taurine concentrations can be interpreted as a beneficial adaptation associated with a more economical management of muscle taurine resources. The exact mechanism remains to be explained as there is a variety of factors affecting the release of taurine from the muscle including changes in the electrophysical properties of the muscle membrane, its co-release with water to maintain plasma volume, and Ca^2+^ homeostasis [56,57].

### 4.4. Practical Perspective

Due to the scarcity of long-term studies, clinical conclusions and practical guidelines drawn from PFAA profiles are still limited. However, this could prove to be an interesting diagnostic approach for the future. By providing data from a long training cycle in a homogeneous group of high-level athletes using precise, validated analytical methods, our study contributes to filling a gap. The complete set of proteinogenic PFAAs, accompanied by related non-proteinogenic metabolites, can be used in several ways. Changes in the concentration of PFAAs (at rest and, more importantly, in response to exercise) can provide information about the adequacy of metabolic adaptation to specific chronic exercise loads or warn of overtraining. The time structure of sports training is organized into cycles, phases, sub-phases, macro-cycles, and micro-cycles, which have specific goals and adequately tailored exercise stimuli [18]. This results in a different magnitude and direction of adaptation response in each period. The PFAA levels can complement the assessment of training status and allow for more a precise planning and control of the training volume and intensity. The (pre-)competition phase seems to be critical and most diagnostically sensitive. Further research is needed to establish reference levels of PFAAs for athletes, especially non-proteinogenic PFAAs, depending on the sport practiced, sex, and training phase. The values may differ significantly from those found in the healthy general population [17]. Moreover, it cannot be ruled out that the levels of some PFAAs in athletes may overlap with those observed in diseased individuals, such as cancer patients [58], which requires data to distinguish between physiological and pathological states (as is the case, for example, with the “athlete’s heart” phenomenon). Finally, our study may stimulate research in the area of nutrition and supplementation, even though such data were not included or analyzed here. Current recommendations from the International Society of Sports Nutrition for endurance athletes include adequate carbohydrate intake with added protein to compensate for muscle (myofibrillar) damage, support recovery, and maintain a balance between protein breakdown and synthesis [59,60]. However, non-proteinogenic amino acid supply and training phases are not included. This may be important because endurance sports have a very high turnover of amino acids in the metabolic processes, which are not related to building muscle mass, but to energy metabolism (such as mitochondrial activity).

### 4.5. Limitations 

The results of our study apply only to highly trained endurance athletes and cannot be used to interpret the levels of PFAAs in athletes of other specialties (e.g., speed-power sports) or in the general population. This study did not include female athletes, who may have a different metabolic response to training and exercise. Also, the concentrations in plasma or whole blood do not necessarily reflect the PFAA levels in organs. Therefore, the net release and uptake of PFAAs by different organs, especially skeletal muscle, is not known and information on the actual production and consumption of amino acids is not available. Unfortunately, sophisticated invasive techniques (e.g., muscle biopsies or arteriovenous tracers) were basically inapplicable in athletes during acute exercise because of muscle damage that would interrupt normal training and competition, but also technically because of the short duration of the progressive exercise we used, frequent sampling, and unsteady blood flow. Another limitation is the lack of control over the athletes’ diet and supplementation, which does not allow us to determine to what extent this factor complemented the effects of training. 

## 5. Conclusions

Our study shows that a long cycle of specialized training over several months causes significant changes in the PFAA profile of highly trained endurance athletes. There is a change in the pattern and magnitude of the response to progressive exercise between the major training phases in which training loads are modified, with the competition phase showing the most distinct metabolic picture. The concentration of most PFAAs and related metabolites, both proteinogenic and non-proteinogenic, is altered by long-term training stimuli. The modified PFAA profile may indicate adaptations in energy metabolism pathways that are critical for training status and endurance performance. The potentially affected pathways include, but are not limited to, the TCA cycle, oxidative phosphorylation, neutralization of toxic ammonia, the purine nucleotide cycle, or buffering of intracellular H^+^ ions. Importantly, the observed changes are essentially an expression of normal adaptation and optimization of metabolic processes, as confirmed by the levels of PFAAs and biomarkers of overtraining. The PFAA levels obtained may provide comparative data for further studies in athletes undergoing long training cycles or in other specific populations and cohorts. 

## Figures and Tables

**Table 1:** Descriptive characteristics in the four subsequent training phases in endurance athletes.

	Training Phases				ANOVA
	Transition	General	Specific	Competition	*p*-Value (*η*^2^)
Weight (kg)	75.2 ± 8.9	73.5 ± 8.1	73.7 ± 7.9	73.7 ± 7.7	0.052 (0.28)
Body Mass Index (kg·m^−2^)	22.7 ± 2.4	22.2 ± 2.2	22.3 ± 2.0	22.3 ± 1.9	0.058 (0.27)
Fat Mass (kg)	12.2 ± 3.1	10.1 ± 2.4 ^T^	10.0 ± 2.1 ^T^	10.1 ± 1.9 ^T^	0.001 (0.54)
Fat Mass (%)	16.0 ± 2.9	13.6 ± 2.4 ^T^	13.4 ± 2.0 ^T^	13.7 ± 1.7 ^T^	<0.001 (0.54)
Skeletal Muscle Mass (kg)	32.7 ± 4.0	32.4 ± 3.5	32.8 ± 3.7	32.8 ± 3.5	0.570 (0.06)
Skeletal Muscle Mass (%)	43.5 ± 1.7	44.2 ± 1.5	44.4 ± 1.5 ^T^	44.5 ± 1.1 ^T^	0.013 (0.30)
White Blood Cells (10^9^·L^−1^)	4.9 ± 0.8	4.6 ± 1.1	5.4 ± 1.0	4.9 ± 0.9	0.161 (0.16)
Red Blood Cells (10^12^·L^−1^)	4.9 ± 0.4	5.0 ± 0.4	4.9 ± 0.4	4.8 ± 0.4	0.177 (0.15)
Hemoglobin (mmol·L^−1^)	9.4 ± 0.4	9.3 ± 0.6	9.2 ± 0.5	8.6 ± 0.6 ^T,G,S^	0.002 (0.52)
Hematocrit (%)	42.4 ± 2.4	42.9 ± 2.4	43.2 ± 2.6	42.4 ± 2.7	0.652 (0.05)
Creatine Kinase (U·L^−1^)	164 ± 118	184 ± 89	190 ± 149	235 ± 138	0.480 (0.08)
$\dot{V}$O_2max_ (L·min^−1^)	4.84 ± 0.75	4.84 ± 0.55	4.98 ± 0.67	5.04 ± 0.7	0.149 (0.16)
$\dot{V}$O_2max_ (L·min^−1^·kg weight ^−1^)	64.1 ± 3.8	66.0 ± 2.9	67.5 ± 3.9	68.2 ± 4.1 ^T^	0.032 (0.25)
$\dot{V}$O_2max_ (L·min^−1^·kg SMM^−1^)	148 ± 11	150 ± 10	152 ± 12	153 ± 11	0.281 (0.12)
Maximum Heart Rate (bpm)	191 ± 7	188 ± 7	190 ± 6	188 ± 7 ^T^	0.032 (0.30)

Abbreviations: SMM, skeletal muscle mass; $\dot{V}$O_2max_, maximum oxygen uptake; ^T^ significantly different from the transition phase; ^G^ significantly different from the general phase; ^S^ significantly different from the specific phase.

**Table 2:** Essential plasma free amino acid (PFAA) concentrations in endurance athletes at rest, under exercise-indued exhaustion, and during post-exercise recovery in the four main training phases of a 9-month cycle. All values are expressed in μmol∙L^−1^. Significant ANOVA effects of the training phase, exercise stage, and their interaction are shown in bold. Significant post-hoc differences from a training phase or exercise stage: T—transition; G—general; S—specific; C—competition; R—rest; E—exhaustion; P_15_—15 min post exercise; P_30_—30 min post exercise.

	Training Phase	Rest	Exhaustion	Post Exercise		ANOVA Effects *p*-Value (*η*^2^)*,* Post-Hoc		
				15th min	30th min	Training Phase	Exercise Stage	Interaction
Histidine	T	82.6 ± 21.9	77.0 ± 14.0	90.8 ± 22.0	81.5 ± 35.9	0.109 (0.14)	**<0.001 (0.18)** E < P_15_,P_30_	0.247 (0.09)
	G	82.2 ± 10.9	84.8 ± 18.9	89.3 ± 14.3	95.4 ± 22.7			
	S	78.1 ± 15.8	75.2 ± 13.3	90.6 ± 22.3	86.0 ± 16.2			
	C	79.0 ± 13.8	66.7 ± 9.1	74.0 ± 12.2	76.1 ± 7.3			
	C	137 ± 45	96 ± 12 ^R^	102 ± 17 ^R^	99 ± 14 ^R^			
Lysine	T	191 ± 34	231 ± 54	177 ± 41 ^E^	185 ± 27 ^E^	0.671 (0.04)	**<0.001 (0.19)** R > P_15_,P_30_ E > P_15_,P_30_	**0.006 (0.20)**
	G	202 ± 35	210 ± 56	189 ± 27	197 ± 36			
	S	197 ± 26	185 ± 28	184 ± 25	178 ± 27			
	C	223 ± 47	172 ± 61	172 ± 33 ^R^	177 ± 30 ^R^			
Methionine	T	25.3 ± 6.3	29.8 ± 8.8	26.2 ± 9.0	26.3 ± 6.2	0.516 (0.06)	0.572 (0.02)	**0.002 (0.21)**
	G	28.6 ± 10.2	29.3 ± 9.0	29.3 ± 5.3	31.6 ± 8.5			
	S	26.1 ± 4.5	24.4 ± 5.1	27.0 ± 5.9	27.2 ± 6.7			
	C	32.8 ± 7.3	25.3 ± 4.3 ^R^	26.8 ± 5.1	27.2 ± 4.4			
Phenylalanine	T	59.4 ± 11.6	54.1 ± 10.5	59.6 ± 16.0	59.9 ± 11.3	0.230 (0.10)	**0.001 (0.14)** E < R,P_30_	**0.015 (0.15)**
	G	64.3 ± 12.1	63.3 ± 11.7	64.6 ± 7.2	69.1 ± 12.6			
	S	62.5 ± 6.4	59.8 ± 8.7	64.7 ± 8.8	65.1 ± 9.1			
	C	71.6 ± 11.3	57.5 ± 4.8 ^R^	59.9 ± 6.1 ^R^	61.8 ± 4.1			
Threonine	T	131 ± 28	108 ± 17	113 ± 50	129 ± 29	0.168 (0.12)	**<0.001 (0.43)** R > E,P_15_ E < P_15_ P_30_	0.188 (0.10)
	G	140 ± 31	112 ± 44	130 ± 22	144 ± 31			
	S	125 ± 14	101 ± 13	117 ± 22	117 ± 19			
	C	136 ± 32	88 ± 36	109 ± 26	117 ± 24			
Tryptophan	T	50.0 ± 10.5	34.7 ± 6.6	44.2 ± 12.6	46.8 ± 10.1	0.320 (0.08)	**<0.001 (0.61)** R > E, P_15_,P_30_ E < P_15_,P_30_ P_15_ < P_30_	0.297 (0.08)
	G	51.2 ± 7.1	40.0 ± 7.0	44.4 ± 6.0	51.9 ± 9.1			
	S	51.3 ± 10.6	37.4 ± 8.7	44.6 ± 8.8	49.9 ± 9.0			
	C	50.6 ± 8.0	33.8 ± 4.8	38.3 ± 6.1	43.5 ± 5.8			
Isoleucine	T	61.0 ± 26.8	53.4 ± 12.8	60.2 ± 16.1	58.1 ± 17.1	0.527 (0.05)	**0.011 (0.12)** R > E,P_30_	**0.009 (0.21)**
	G	60.0 ± 13.8	58.3 ± 8.4	60.2 ± 7.0	61.7 ± 5.2			
	S	52.7 ± 9.4	50.9 ± 9.9	55.8 ± 9.7	53.8 ± 12.8			
	C	76.4 ± 29.4 ^S^	52.9 ± 9.3 ^R^	54.8 ± 10.3 ^R^	52.7 ± 9.5 ^R^			
Leucine	T	105 ± 40	84 ± 45	113 ± 27	111 ± 27	0.955 (0.01)	**0.030 (0.09)** R > E	**0.002 (0.23)**
	G	110 ± 21	107 ± 17	112 ± 14	115 ± 14			
	S	95 ± 34	102 ± 19	113 ± 16	108 ± 21			
	C	137 ± 45	96 ± 12 ^R^	102 ± 17 ^R^	99 ± 14 ^R^			
Valine	T	241 ± 44	211 ± 26	243 ± 55	242 ± 51	0.328 (0.08)	**<0.001 (0.25)** R > E E < P_15_,P_30_	**0.002 (0.19)**
	G	251 ± 28	237 ± 33	251 ± 22	264 ± 27			
	S	249 ± 26	227 ± 31	257 ± 36	255 ± 39			
	C	285 ± 114	233 ± 32 ^R^	248 ± 40 ^R^	256 ± 37 ^R^			
Total BCAAs	T	407 ± 107	367 ± 53	417 ± 96	411 ± 93	0.681 (0.04)	**<0.001 (0.18)** R > E E < P_15_,P_30_	**0.002 (0.22)**
	G	420 ± 58	402 ± 56	423 ± 39	441 ± 39			
	S	405 ± 48	380 ± 58	426 ± 60	417 ± 71			
	C	516 ± 147	381 ± 50 ^R^	405 ± 66 ^R^	408 ± 58 ^R^			
Total essential PFAAs	T	856 ± 322	749 ± 374 ^R^	937 ± 209 ^R^	949 ± 170 ^R^	0.587 (0.05)	**<0.001 (0.16)** R > E,P_15_ E < P_30_	**0.006 (0.18)**
	G	898 ± 323	951 ± 157	879 ± 299	1030 ± 136			
	S	946 ± 102	693 ± 351	953 ± 131	940 ± 133			
	C	1109 ± 225	851 ± 84	804 ± 296	910 ± 108			

**Table 3:** Non-essential plasma free amino acid (PFAA) concentrations in endurance athletes at rest, under exercise-induced exhaustion, and during post-exercise recovery in the four main training phases of a 9-month cycle. All values are expressed in μmol∙L^−1^. Significant ANOVA effects of the training phase, exercise stage, and their interaction are shown in bold. Significant post-hoc differences from a training phase or exercise stage: T—transition; G—general; S—specific; C—competition; R—rest; E—exhaustion; P_15_—15 min post exercise; P_30_—30 min post exercise.

	Training Phase	Rest	Exhaustion	Post Exercise		ANOVA Effects *p*-Value (*η*^2^), Post-Hoc		
				15th min	30th min	Training Phase	Exercise Stage	Interaction
Alanine	T	393 ± 63	477 ± 177	567 ± 99	570 ± 134	0.207 (0.03)	**<0.001 (0.35)** R < E,P_15_, P_30_	0.085 (0.12)
	G	409 ± 75	589 ± 159	594 ± 63	609 ± 109			
	S	401 ± 78	534 ± 98	550 ± 127	513 ± 98			
	C	408 ± 78	509 ± 43	506 ± 50	519 ± 49			
Arginine	T	72.7 ± 15.6	91.4 ± 30.9 ^C^	67.5 ± 16.9 ^E^	68.7 ± 19.0 ^E^	0.155 (0.12)	**0.005 (0.11)** E > P_15_,P_30_	**0.046** **(0.14)**
	G	71.9 ± 17.3	81.0 ± 23.8	68.3 ± 14.6	75.4 ± 16.1			
	S	68.4 ± 12.9	69.4 ± 13.7	67.2 ± 15.3	66.5 ± 18.1			
	C	71.8 ± 19.1	62.6 ± 9.7	59.9 ± 8.7	60.9 ± 13.6			
Asparagine	T	58.5 ± 9.8	51.4 ± 8.9	55.8 ± 13.8	59.4 ± 14.3	0.221 (0.10)	**<0.001 (0.26)** R > E,P_15_	0.194 (0.10)
	G	63.6 ± 22.5	49.1 ± 20.6	62.5 ± 11.9	68.0 ± 14.6			
	S	62.4 ± 10.9	54.1 ± 9.4	58.1 ± 10.3	59.6 ± 12.5			
	C	62.1 ± 9.0	47.4 ± 6.0	50.3 ± 6.8	53.8 ± 7.3			
Aspartate	T	3.14 ± 1.24	5.63 ± 3.42 ^R^	3.59 ± 1.30 ^E^	3.39 ± 1.25 ^E^	**0.019 (0.22)** T < C	**0.006 (0.11)** R < E,P_15_	**0.011** **(0.17)**
	G	2.66 ± 0.99	3.12 ± 1.26 ^T^	3.34 ± 1.28	2.85 ± 0.76			
	S	2.20 ± 1.06	2.57 ± 1.06 ^T^	3.45 ± 1.21	2.73 ± 1.10			
	C	2.30 ± 1.47	2.43 ± 1.42 ^T^	3.06 ± 1.45	2.64 ± 1.63			
Cystine	T	40.5 ± 9.1	36.3 ± 8.7	48.5 ± 12.9	47.4 ± 9.6	**0.042 (0.18)**	**<0.001 (0.40)** R,E < P_15_,P_30_	0.346 (0.08)
	G	47.5 ± 7.2	48.3 ± 10.8	56.0 ± 9.0	59.6 ± 11.8			
	S	43.8 ± 10.2	41.0 ± 9.0	50.9 ± 13.5	51.7 ± 13.3			
	C	44.8 ± 9.9	39.3 ± 5.2	45.5 ± 7.3	47.4 ± 6.5			
Glutamate	T	34.7 ± 20.0	23.9 ± 11.7	42.8 ± 13.2 ^E^	60.9 ± 21.8 ^R,E,P15^	**0.006 (0.27)** T > C	**<0.001 (0.60)** R,E < P_15_,P_30_	**0.004** **(0.18)**
	G	24.3 ± 11.3	20.4 ± 9.2	47.1 ± 22.5 ^R,E^	43.6 ± 13.5 ^R,E^			
	S	17.1 ± 5.3	23.5 ± 14.1	40.4 ± 17.1 ^R,E^	44.7 ± 17.3 ^R,E^			
	C	18.8 ± 9.3	12.7 ± 5.8	29.9 ± 9.8 ^E^	30.5 ± 10.9 ^T,E^			
Glutamine	T	670 ± 115	656 ± 127	715 ± 198	690 ± 176	**0.008 (0.26)** G > C	0.289 (0.03)	0.268 (0.09)
	G	693 ± 127	766 ± 166	709 ± 147	802 ± 151			
	S	649 ± 140	660 ± 156	666 ± 132	676 ± 107			
	C	617 ± 90	549 ± 83	507 ± 191	545 ± 202			
Glycine	T	192 ± 31	173 ± 28	165 ± 43	172 ± 43	0.121 (0.13)	**<0.001 (0.19)** R > P^15^,P^30^ E > P^15^	0.499 (0.07)
	G	192 ± 40	197 ± 53	173 ± 26	189 ± 31			
	S	173 ± 34	166 ± 23	159 ± 29	156 ± 25			
	C	183 ± 20	162 ± 20	149 ± 23	145 ± 52			
Proline	T	218 ± 62	184 ± 46	212 ± 61	233 ± 69	0.727 (0.03)	**<0.001 (0.35)** R > E; E < P_15_,P_30_ P_15_ < P_30_	0.085 (0.12)
	G	216 ± 42	201 ± 49	195 ± 75	249 ± 60			
	S	222 ± 47	186 ± 37	219 ± 47	224 ± 43			
	C	229 ± 61	168 ± 41	169 ± 74	209 ± 50			
Serine	T	97.4 ± 18.4	95.5 ± 26.5	96.2 ± 22.6	98.5 ± 19.5	**<0.001 (0.35)** T, G > C	**0.001 (0.15)** R > E E < P_30_	0.305 (0.08)
	G	93.7 ± 35.0	90.1 ± 33.1	101.8 ± 16.1	113.5 ± 16.5			
	S	96.0 ± 14.3	82.4 ± 13.8	96.3 ± 13.2	97.5 ± 15.0			
	C	90.9 ± 15.8	67.4 ± 11.4	77.2 ± 16.9	80.0 ± 11.4			
Tyrosine	T	68.3 ± 16.5	66.0 ± 14.1	70.1 ± 20.4	71.8 ± 20.9	0.862 (0.02)	**<0.001 (0.17)** R > E E < P_30_	**0.001 ** **(0.21)**
	G	72.4 ± 15.3	71.3 ± 19.0	72.7 ± 10.9	77.9 ± 17.1			
	S	73.7 ± 16.1	66.1 ± 12.9	73.5 ± 17.7	63.2 ± 25.1			
	C	85.8 ± 29.3	68.1 ± 15.9 ^R^	62.2 ± 23.9 ^R^	73.5 ± 17.7 ^R^			
Total non-essen- tial PFAAs	T	1849 ± 296	1716 ± 637	2043 ± 457	2075 ± 438	**0.014 (0.23)** G > C	**0.003 (0.13)** R < P_30_ E < P_30_	0.251 (0.09)
	G	1894 ± 285	2134 ± 454	2100 ± 236	2290 ± 337			
	S	1809 ± 298	1696 ± 632	1983 ± 339	1820 ± 644			
	C	1670 ± 583	1688 ± 151	1730 ± 194	1840 ± 163			

**Table 4:** The concentration of non-proteinogenic plasma free amino acids and related metabolites at rest, under exercise-induced exhaustion, and during post-exercise recovery in the four main training phases of a 9-month cycle. All values are expressed in μmol∙L^−1^. Significant ANOVA effects of the training phase, exercise stage, and their interaction are shown in bold. Significant post-hoc differences from a training phase or exercise stage: T—transition; G—general; S—specific; C—competition; R—rest; E—exhaustion; P_15_—15 min post exercise; P_30_—30 min post exercise.

	Training Phase	Rest	Exhaustion	Post Exercise		ANOVA Effects *p*-Value (*η*^2^)*,* Post-Hoc		
				15th min	30th min	Training Phase	Exercise Stage	Interaction
1-Methylhistidi-ne	T	10.9 ± 9.4	9.2 ± 8.1	10.1 ± 8.6	9.8 ± 8.2	0.553 (0.05)	**<0.001** **(0.16)** R > E,P_15_,P_30_	0.107 (0.12)
	G	10.6 ± 9.3	10.6 ± 9.9	10.1 ± 9.0	10.9 ± 10.1			
	S	7.9 ± 7.8	7.0 ± 7.3	6.6 ± 5.9	7.0 ± 6.8			
	C	14.2 ± 9.6	11.1 ± 7.1	11.4 ± 7.2	12.0 ± 7.9			
3-Methylhisti- dine	T	5.02 ± 1.24	4.77 ± 1.05	5.18 ± 1.35	5.27 ± 1.06	0.738 (0.03)	**<0.001** **(0.15)** E < P_15_,P_30_	0.320 (0.08)
	G	5.16 ± 1.17	5.31 ± 1.62	5.34 ± 1.00	5.72 ± 1.33			
	S	4.89 ± 1.56	4.65 ± 1.30	5.18 ± 1.52	5.10 ± 1.57			
	C	5.65 ± 1.20	5.13 ± 1.04	5.35 ± 1.32	5.70 ± 1.10			
Aminoadipic acid	T	0.74 ± 0.23	0.82 ± 0.29	0.98 ± 0.33 ^R^	0.88 ± 0.23	0.101 (0.14)	**<0.001 (0.34)** R < E,P_15_,P_30_ E < P_15_ P_15_ > P_30_	**0.023** **(0.15)**
	G	0.82 ± 0.22	1.08 ± 0.46 ^R^	1.15 ± 0.23 ^R^	1.03 ± 0.25			
	S	0.79 ± 0.25	1.02 ± 0.34	1.11 ± 0.28 ^R^	0.96 ± 0.21			
	C	1.11 ± 0.32	1.12 ± 0.25	1.15 ± 0.19	1.08 ± 0.22			
Aminobutyric acid	T	22.0 ± 7.0	17.5 ± 7.5	20.4 ± 7.5	21.7 ± 8.2	0.883 (0.02)	**<0.001** **(0.57)** R > E,P_15_ E < P_15_,P_30_	0.103 (0.12)
	G	22.3 ± 7.0	18.4 ± 6.1	21.6 ± 5.6	22.7 ± 7.2			
	S	22.3 ± 6.8	18.1 ± 6.1	20.8 ± 6.8	20.8 ± 6.1			
	C	22.6 ± 7.5	15.6 ± 4.4	18.5 ± 5.4	19.6 ± 5.6			
β-Aminoisobu- tyric acid	T	1.42 ± 0.56	1.98 ± 0.76 ^R^	1.96 ± 0.98 ^R^	1.89 ± 0.72 ^R^	0.889 (0.02)	**<0.001** **(0.14)** R < E	**0.007** **(0.17)**
	G	1.63 ± 0.58	1.93 ± 0.68	1.77 ± 0.73	1.97 ± 0.79			
	S	2.03 ± 0.83	2.06 ± 0.84	1.93 ± 0.77	1.88 ± 0.77			
	C	1.95 ± 0.67	2.11 ± 0.84	1.93 ± 0.61	1.91 ± 0.62			
β-Alanine	T	29.9 ± 9.1	27.9 ± 6.0	29.7 ± 6.9	31.8 ± 5.9	**<0.001 (0.47)** C > T,G,S	**0.045** **(0.07)** E < P30	0.162 (0.10)
	G	30.5 ± 9.6	30.9 ± 13.0	28.6 ± 5.9	40.9 ± 19.7			
	S	31.2 ± 7.4	25.6 ± 4.7	34.8 ± 7.2	27.7 ± 6.8			
	C	38.4 ± 9.2	36.2 ± 6.4	40.1 ± 8.2	43.0 ± 9.6			
Citrulline	T	27.7 ± 7.5	25.0 ± 7.7	27.0 ± 10.7	26.3 ± 10.4	0.906 (0.01)	0.053 (0.07)	0.182 (0.10)
	G	29.1 ± 8.9	29.3 ± 9.5	27.8 ± 6.2	28.9 ± 6.2			
	S	27.9 ± 6.6	26.5 ± 6.6	28.1 ± 6.8	26.4 ± 7.0			
	C	33.9 ± 19.1	27.7 ± 9.7	26.4 ± 7.0	25.8 ± 7.5			
Ethanolamine	T	8.8 ± 1.7	11.6 ± 1.5	10.1 ± 1.9	9.8 ± 2.2	0.430 (0.07)	**<0.001 (0.64)** R < E,P_15_,P_30_ E > P_15_,P_30_ P_15_ < P_30_	0.338 (0.08)
	G	8.1 ± 0.9	12.0 ± 1.6	10.9 ± 1.8	9.8 ± 1.7			
	S	8.7 ± 1.8	11.5 ± 2.4	10.8 ± 1.9	9.3 ± 1.6			
	C	9.5 ± 1.6	12.7 ± 1.7	11.2 ± 1.5	10.5 ± 1.3			
Hydroxyproli-ne	T	11.7 ± 7.0	8.8 ± 6.2	10.3 ± 6.1	11.6 ± 7.6	0.563 (0.05)	**<0.001 (*0.47*)** R > E,P_15_ E < P_15_,P_30_ P_15_ > P_30_	0.794 (0.04)
	G	12.3 ± 6.8	9.9 ± 5.2	11.0 ± 5.5	12.5 ± 6.4			
	S	10.1 ± 6.3	7.7 ± 4.9	9.3 ± 5.4	9.6 ± 6.2			
	C	9.6 ± 4.9	6.8 ± 3.7	8.1 ± 4.9	8.4 ± 3.9			
Ornithine	T	61.1 ± 16.9	54.5 ± 13.7	59.4 ± 25.8	56.8 ± 19.0	0.680 (0.04)	**<0.001** **(0.20)** R > E,P_15_,P_30_	0.172 (0.10)
	G	67.7 ± 16.7	63.6 ± 12.5	60.0 ± 11.7	60.3 ± 9.8			
	S	60.7 ± 12.8	52.7 ± 11.1	56.2 ± 14.2	51.8 ± 12.1			
	C	69.6 ± 24.3	59.0 ± 19.1	44.1 ± 15.6	54.1 ± 14.5			
Phosphoetha- nolamine	T	3.62 ± 3.04	3.55 ± 2.99	3.97 ± 2.14	3.92 ± 2.60	**0.037 (0.19)**	**0.026** **(0.08)** R < P15	0.181 (0.10)
	G	3.12 ± 1.62	4.35 ± 2.46	4.24 ± 2.31	4.03 ± 2.17			
	S	2.43 ± 1.34	3.70 ± 1.82	4.28 ± 2.34	3.85 ± 2.31			
	C	2.09 ± 1.01	2.05 ± 0.55	1.98 ± 0.99	1.53 ± 0.78			
Sarcosine	T	2.31 ± 0.81	1.85 ± 0.61	2.20 ± 0.96	2.47 ± 1.03 ^E^	**<0.001 (0.41)** T,G,S < C	**<0.001** **(0.33)** R > E,P_15_ E < P_15_,P_30_	**0.017** **(0.15)**
	G	2.39 ± 0.86	2.15 ± 0.95	2.46 ± 1.03	2.43 ± 0.79			
	S	2.71 ± 0.94	2.36 ± 0.88	2.59 ± 0.85	2.68 ± 1.30			
	C	4.46 ± 0.83 ^T,G,S^	3.63 ± 0.91 ^T,R^	3.80 ± 0.82 ^T,R^	4.01 ± 0.97 ^T,G^			
Taurine	T	68.7 ± 43.6	63.6 ± 37.8	71.1 ± 27.8	76.2 ± 34.1	**0.008 (0.25)** T > C G > C	*0.056 * (0.07)	0.338 (0.08)
	G	60.4 ± 21.7	70.9 ± 25.7	70.7 ± 16.4	70.0 ± 15.5			
	S	53.7 ± 19.6	65.0 ± 20.3	73.8 ± 22.9	65.7 ± 16.5			
	C	42.9 ± 10.9	42.5 ± 6.9	45.0 ± 13.0	40.2 ± 8.0			
Total non-proteino- genic PFAAs	T	254 ± 69	231 ± 68	252 ± 67	258 ± 69	0.467 (0.06)	0.109 (0.05)	0.069 (0.13)
	G	254 ± 41	260 ± 49	256 ± 23	271 ± 27			
	S	235 ± 48	228 ± 45	255 ± 48	233 ± 44			
	C	256 ± 46	226 ± 28	228 ± 34	210 ± 71			

**Table 5:** Training status biomarkers in endurance athletes at rest, under exercise-induced exhaustion, and during post-exercise recovery in the four main training phases of a 9-month cycle. All values are expressed in μmol∙L^−l^. Significant ANOVA effects of the training phase, exercise stage, and their interaction are shown in bold. Significant post-hoc differences from a training phase or exercise stage: T—transition; G—general; S—specific; C—competition; R—rest; E—exhaustion; P_15_—15 min post exercise; P_30_—30 min post exercise.

	Training Phase	Rest	Exhaustion	Post Exercise		ANOVA Effects *p*-Value (*η*^2^)*,* Post-Hoc		
				15th min	30th min	Training Phase	Exercise Stage	Interaction
Ammonia (μmol·L^−1^)	T	26.4 ± 4.3	80.0 ± 8.2 ^R^	55.9 ± 6.5 ^R,E^	35.2 ± 5.0 ^R,E,P15^	**<0.001 (0.51)** T > S,C G > C	**<0.001 (0.94)** R < E,P_15_,P_30_ E > P_15_,P_30_ P_15_ > P_30_	**0.005** **(0.21)**
	G	26.3 ± 5.1	74.6 ± 11.1 ^R^	48.5 ± 5.8 ^R,E^	32.1 ± 2.8 ^E,P15^			
	S	23.1 ± 3.1	65.7 ± 11.6 ^R^	41.1 ± 6.4 ^R,E^	27.9 ± 3.2 ^E,P15^			
	C	19.6 ± 3.2	60.3 ± 11.4 ^R^	40.5 ± 5.1 ^R,E^	27.8 ± 6.4 ^E,P15^			
Lactate (mmol·L^−1^)	T	1.16 ± 0.32	11.32 ± 1.81	8.32 ± 2.29	5.17 ± 1.71	0.226 (0.10)	**<0.001 (0.95)** R < E,P_15_,P_30_ E > P_15_,P3_0_ P_15_ > P_30_	0.374 (0.08)
	G	1.01 ± 0.18	10.30 ± 1.52	6.61 ± 1.77	4.30 ± 1.78			
	S	0.90 ± 0.18	10.60 ± 2.05	6.60 ± 2.16	3.90 ± 1.63			
	C	0.91 ± 0.31	10.47 ± 1.70	6.72 ± 1.80	4.04 ± 1.34			
Phenylalani- ne: Tyrosine ratio	T	0.89 ± 0.16	0.85 ± 0.19	0.88 ± 0.18	0.87 ± 0.18	0.961 (0.17)	0.812 (0.01)	**0.041** **(0.14)**
	G	0.90 ± 0.12	0.91 ± 0.11	0.90 ± 0.12	0.90 ± 0.10			
	S	0.95 ± 0.33	0.98 ± 0.25	0.99 ± 0.35	0.98 ± 0.25			
	C	0.90 ± 0.21	0.89 ± 0.21	0.88 ± 0.18	0.88 ± 0.21			
Glutamine: Glutamate ratio	T	26.5 ± 16.0	34.0 ± 17.8	18.6 ± 9.0	14.0 ± 9.4	0.293 (0.09)	**<0.001 (0.53)** R < E,P_15_,P_30_ E > P_15_,P_30_	0.275 (0.09)
	G	35.5 ± 19.9	46.3 ± 28.9	19.1 ± 11.2	23.3 ± 20.2			
	S	41.0 ± 14.5	37.7 ± 19.0	18.9 ± 7.8	17.4 ± 6.8			
	C	38.1 ± 13.9	49.7 ± 18.2	18.3 ± 7.6	20.7 ± 12.4			

## Data Availability

The original raw data analyzed in this study are included in the Supplementary Materials; further inquiries can be directed to the corresponding author.

## Supplementary Materials

The following supporting information can be downloaded at: https://www.mdpi.com/article/10.3390/metabo14070353/s1, Supplementary File S1: Original raw data.

## Appendix A

### The Protocol of Plasma Free Amino Acids Analysis

Each 40-μL plasma sample was transferred to an Eppendorf tube and 10 μL of 10% sulfosalicylic acid was added to precipitate proteins present in the plasma, then the contents were mixed and centrifuged (10,000× *g* for 2 min). The supernatant was then transferred to a new tube and mixed with 40 μL of borate buffer. A 10-μL aliquot of the resulting solution was then labeled with the aTRAQ reagent Δ8 solution (5 μL), mixed, centrifuged, and incubated at room temperature for 30 min. The labeling reaction was then stopped by adding 5 μL of 1.2% hydroxylamine, mixing, and incubating at room temperature for 15 min. Then, 32 μL of the internal standard solution was then added and the content was mixed. The internal standard solution contained the same amino acids labeled with the aTRAQ reagent Δ0. Thus, each amino acid assayed had a corresponding internal standard. In the next step, the sample was evaporated in a vacuum concentrator (miVac Duo Concentrator, Genevac, Stone Ridge, NY, USA) for 15 min to reduce the volume to ~20 μL. The residue was then diluted with 20 μL of water, mixed, and transferred to an autosampler vial with an insert. Norleucine and norvaline, two non-proteinogenic amino acids, were used to evaluate the labeling efficiency and recovery. The corresponding internal standards were also included in the internal standard solution.

Analyses were performed using the 1260 Infinity liquid chromatography instrument (Agilent Technologies, Santa Clara, CA, USA) coupled to the 4000 QTRAP mass spectrometer (Sciex, Framingham, MA, USA). The mass spectrometer was equipped with an electrospray ionization source and three quadrupoles to allow quantitative analysis of the target amino acids. Chromatographic separation was performed using the Sciex C18 (5 μm, 4.6 mm × 150 mm) chromatography column. The mobile phase flow rate was maintained at 800 μL∙min-1. The method used two mobile phases, water (phase A) and methanol (phase B), both with the addition of 0.1% formic acid and 0.01% heptafluorobutyric acid. The analysis time was 18 min, and during this time, the chromatographic separation was performed with the following gradient elution: from 2% to 40% of phase B from 0 to 6 min, then maintained at 40% of phase B for 4 min, increased to 90% of phase B until the 11th minute, maintained at this ratio of phases for 1 min, then decreased to 2% of phase B, and finally maintained at 2% of phase B from the 13th to 18th minutes. The injection volume was preset at 2 μL and the separation temperature at 50 °C. The following ion source settings were used: curtain gas = 20 psig; ion spray voltage = 4500 V; ion source temperature = 600 °C; ion source gas 1 = 60 psig, ion source gas 2 = 50 psig. The mass spectrometer was operated in positive ionization mode with the following parameters: entrance potential = 10 V; declustering potential = 30 V; collision cell exit potential = 5 V; collision energy = 30 eV (50 eV in the case of 7 compounds); nitrogen was used as the collision gas. Amino acids were quantified in the scheduled multiple reaction monitoring (sMRM) mode, as shown in Table A1). This mode provided high specificity and sensitivity for quantitative analyses. Data were collected and processed using Analyst 1.5 software (Sciex, Framingham, MA, USA).

**Table A1:** Multiple reaction monitoring (MRM) transitions for 42 amino acids and related metabolitesanalyzed using LC-MS/MS-base methodology and their corresponding internal standards with the applied collision energy values and limit of quantitation values achieved by the methodology used.

No	Amino Acid	Abbrev.	MRM Transition (Q1 → Q3)		Collision Energy (eV)	Limit of Quantitation (μmol∙L^−1^)
			Analyte	Internal Std.		
1	1-methylhistidine	1MHis	318.2 → 121.1	310.2 → 113.1	30	0.2
2	3-methylhistidine	3MHis	318.2 → 121.1	310.2 → 113.1	30	0.2
3	alanine	Ala	238.2 → 121.1	230.2 → 113.1	30	0.2
4	anserine	Ans	389.2 → 121.1	381.2 → 113.1	30	0.5
5	arginine	Arg	323.2 → 121.1	315.2 → 113.1	30	0.5
6	argininosuccinic acid	Asa	439.2 → 121.1	431.2 → 113.1	50	1.0
7	asparagine	Asn	281.2 → 121.1	273.2 → 113.1	30	0.5
8	aspartic acid	Asp	282.1 → 121.1	274.1 → 113.1	30	0.1
9	carnosine	Car	375.2 → 121.1	367.2 → 113.1	30	0.5
10	citrulline	Cit	324.2 → 121.1	316.2 → 113.1	30	0.5
11	cystathionine	Cth	519.3 → 121.1	503.3 → 113.1	50	0.5
12	cystine	Cys	537.2 → 121.1	521.2 → 113.1	50	1.0
13	ethanolamine	EtN	210.2 → 121.1	202.2 → 113.1	30	0.5
14	glutamic acid	Glu	296.2 → 121.1	288.2 → 113.1	30	0.5
15	glutamine	Gln	295.2 → 121.1	287.2 → 113.1	30	0.5
16	glycine	Gly	224.1 → 121.1	216.1 → 113.1	30	1.0
17	histidine	His	304.2 → 121.1	296.2 → 113.1	30	0.5
18	homocitrulline	Hcit	338.2 → 121.1	330.2 → 113.1	30	0.2
19	homocystine	Hcy	565.3 → 121.1	549.3 → 113.1	50	0.5
20	hydroxyproline	Hyp	280.1 → 121.1	272.1 → 113.1	30	0.2
21	isoleucine	Ile	280.2 → 121.1	272.2 → 113.1	30	0.5
22	leucine	Leu	280.2 → 121.1	272.2 → 113.1	30	0.5
23	lysine	Lys	443.3 → 121.1	427.3 → 113.1	50	0.5
24	methionine	Met	298.2 → 121.1	290.2 → 113.1	30	0.1
25	ornithine	Orn	429.3 → 121.1	413.3 → 113.1	50	0.5
26	phenylalanine	Phe	314.2 → 121.1	306.2 → 113.1	30	0.2
27	phosphoethanolamine	PEtN	290.1 → 121.1	282.1 → 113.1	30	0.5
28	phosphoserine	PSer	334.1 → 121.1	326.1 → 113.1	30	0.5
29	proline	Pro	264.2 → 121.1	256.2 → 113.1	30	0.1
30	sarcosine	Sar	238.2 → 121.1	230.2 → 113.1	30	0.2
31	serine	Ser	254.2 → 121.1	246.2 → 113.1	30	0.5
32	taurine	Tau	274.1 → 121.1	266.1 → 113.1	30	0.5
33	threonine	Thr	268.2 → 121.1	260.2 → 113.1	30	0.2
34	tryptophan	Trp	353.2 → 121.1	345.2 → 113.1	30	0.1
35	tyrosine	Tyr	330.2 → 121.1	322.2 → 113.1	30	0.5
36	valine	Val	266.2 → 121.1	258.2 → 113.1	30	0.2
37	α-aminoadipic acid	Aad	310.2 → 121.1	302.2 → 113.1	30	0.2
38	α-amino-n-butyric acid	Abu	252.2 → 121.1	244.2 → 113.1	30	0.2
39	β-alanine	bAla	238.2 → 121.1	230.2 → 113.1	30	0.5
40	β-aminoisobutyric acid	bAib	252.2 → 121.1	244.2 → 113.1	30	0.2
41	γ-amino-n-butyric acid	GABA	252.2 → 121.1	244.2 → 113.1	30	0.05
42	δ-hydroxylysine	Hyl	459.3 → 121.1	443.3 → 113.1	50	0.5
